# Metabolic Coevolution in the Bacterial Symbiosis of Whiteflies and Related Plant Sap-Feeding Insects

**DOI:** 10.1093/gbe/evv170

**Published:** 2015-09-15

**Authors:** Jun-Bo Luan, Wenbo Chen, Daniel K. Hasegawa, Alvin M. Simmons, William M. Wintermantel, Kai-Shu Ling, Zhangjun Fei, Shu-Sheng Liu, Angela E. Douglas

**Affiliations:** ^1^Department of Entomology, Cornell University; ^2^Boyce Thompson Institute for Plant Research, Cornell University; ^3^USDA-Agricultural Research Service, U.S. Vegetable Laboratory, Charleston, South Carolina; ^4^USDA-Agricultural Research Service, Crop Improvement and Protection Research, Salinas, California; ^5^USDA-Agricultural Research Service, Robert W. Holley Center for Agriculture and Health, Ithaca, New York; ^6^Ministry of Agriculture Key Laboratory of Agricultural Entomology, Institute of Insect Sciences, Zhejiang University, China; ^7^Department of Molecular Biology and Genetics, Cornell University

**Keywords:** amino acid biosynthesis, bacteriocyte, *Bemisia tabaci*, *Hamiltonella*, horizontal gene transfer, *Portiera*

## Abstract

Genomic decay is a common feature of intracellular bacteria that have entered into symbiosis with plant sap-feeding insects. This study of the whitefly *Bemisia tabaci* and two bacteria (*Portiera aleyrodidarum* and *Hamiltonella defensa*) cohoused in each host cell investigated whether the decay of *Portiera* metabolism genes is complemented by host and *Hamiltonella* genes, and compared the metabolic traits of the whitefly symbiosis with other sap-feeding insects (aphids, psyllids, and mealybugs). Parallel genomic and transcriptomic analysis revealed that the host genome contributes multiple metabolic reactions that complement or duplicate *Portiera* function, and that *Hamiltonella* may contribute multiple cofactors and one essential amino acid, lysine. Homologs of the *Bemisia* metabolism genes of insect origin have also been implicated in essential amino acid synthesis in other sap-feeding insect hosts, indicative of parallel coevolution of shared metabolic pathways across multiple symbioses. Further metabolism genes coded in the *Bemisia* genome are of bacterial origin, but phylogenetically distinct from *Portiera*, *Hamiltonella* and horizontally transferred genes identified in other sap-feeding insects. Overall, 75% of the metabolism genes of bacterial origin are functionally unique to one symbiosis, indicating that the evolutionary history of metabolic integration in these symbioses is strongly contingent on the pattern of horizontally acquired genes. Our analysis, further, shows that bacteria with genomic decay enable host acquisition of complex metabolic pathways by multiple independent horizontal gene transfers from exogenous bacteria. Specifically, each horizontally acquired gene can function with other genes in the pathway coded by the symbiont, while facilitating the decay of the symbiont gene coding the same reaction.

## Introduction

Various bacterial lineages have undergone genome reduction, either as an adaptation to environmental conditions (genomic streamlining) or as a nonadaptive consequence of extreme population bottlenecking (genomic decay) (McCutcheon and Moran 2012; Giovannoni et al. 2014; Moran and Bennett 2014). Genomic decay and its evolutionary consequences have been studied particularly in the intracellular bacterial symbionts of hemipteran insects that feed through the life cycle on plant sap (Douglas 2014). Genomic deterioration of these bacteria, whose genomes are 0.1–0.7 Mb, can be attributed to the transmission of small numbers of bacterial cells from the mother insect to the offspring at each insect generation, in some associations for greater than 100 Myr (Moran 1996; McCutcheon and Moran 2012). Because the association is required by both the insect host and the bacterial symbionts, genomic decay is constrained by selection for function. This is particularly evident in relation to the bacterial genetic capacity to synthesize the ten essential amino acids (EAAs) that are deficient in the insect diet of phloem sap and cannot be synthesized de novo by these insects (Akman Gündüz and Douglas 2009; Wilson et al. 2011). The bacterial symbionts have also been implicated in provisioning of some cofactors, notably B vitamins (Douglas 2015). Plant sap feeding through the life cycle has evolved multiple times in one order of insects, Hemiptera, but is otherwise unknown in the animal kingdom.

Genomic approaches are transforming our understanding of the coevolutionary interactions between the bacteria and plant sap-feeding insects. In particular, the metabolic capabilities of the intracellular bacteria can be inferred from their gene complement, and those of the host cells (also known as bacteriocytes) from their gene expression profile. In the several sap-feeding hemipteran groups studied, multiple molecular mechanisms compensating for the genomic decay of the bacterial symbionts have been identified (McCutcheon and Moran 2007; Thomas et al. 2009; McCutcheon and von Dohlen 2011; Macdonald et al. 2012). In particular, aphids, psyllids, and mealybugs (sap-feeding hemipterans of the suborder Sternorrhyncha) display enriched expression of host genes coding reactions mediated by “missing” symbiont genes, and some of these genes are horizontally acquired generally from other bacteria (Nikoh et al. 2010; Hansen and Moran 2011; Poliakov et al. 2011; Husnik et al. 2013; Russell et al. 2013; Sloan et al. 2014). Furthermore, the insects may bear additional symbionts coding genes for some (e.g., in the mealybug *Planococcus citri*) or all (e.g., in the aphid *Cinara cedri*) reactions for certain EAA biosynthetic pathways (Pérez-Brocal et al. 2006; McCutcheon and von Dohlen 2011).

An important caveat to our understanding of the evolutionary history of metabolic coevolution in these symbioses is the lack of information on the fourth major group of sap-feeding sternorrhynchan insects, the whiteflies (superfamily Aleyrodoidea), including globally important crop pests of the *Bemisia tabaci* species complex (De Barro et al. 2011). The whiteflies bear the γ-proteobacterium *Portiera aleyrodidarum* (hereafter *Portiera*) (Thao and Baumann 2004) and one to several additional bacterial species, generically known as secondary symbionts (Gottlieb et al. 2008; Skaljac et al. 2010; Caspi-Fluger et al. 2011). The sequenced genome of *Portiera* in two members of the *B. tabaci* complex (Middle East-Asia Minor 1 and Mediterranean, hereafter MEAM1 and MED) and in *Trialeurodes vaporariorum* reveals that this bacterium has a very small genome (approximately 350 kb) and restricted metabolic capacity (Santos-Garcia et al. 2012; Sloan and Moran 2012, 2013; Jiang et al. 2013). The secondary symbiont in the bacteriocytes of *B. tabaci* MEAM1 is *Candidatus* Hamiltonella defensa (hereafter *Hamiltonella*) (Gottlieb et al. 2008), and its 1.72 Mb genome has recently been sequenced (Rollat-Farnier et al. 2015), but the identity of the secondary symbiont in the bacteriocyte varies, even among closely related members of the *B. tabaci* complex (Gottlieb et al. 2008; Jing et al. 2014; Zchori Fein et al. 2014). Many avenues of research on the symbiosis in whiteflies have been constrained by the very small size of these insects; for example, bacteriocyte dissections from whiteflies are laborious and require great technical skill.

Our research focused on the whitefly *B. tabaci* species MEAM1 housing both *Portiera* and one secondary symbiont *Hamiltonella* in every bacteriocyte (Shan et al. 2014). The specific purpose of this study was 2-fold: 1) To determine whether the fragmented metabolic capability of *Portiera* can be compensated entirely by bacteriocyte function, or may require inputs from the secondary symbiont *Hamiltonella*; and 2) to compare the molecular basis of nutrient exchange in the whitefly symbiosis with previously published analyses of representatives of the other three groups of phloem-feeding sternorrhynchan hemipterans.

## Materials and Methods

### Insect Culture

RNA-Seq (RNA sequencing) was conducted on a *B. tabaci* MEAM1 culture (mtCO1 GenBank accession number GQ332577) collected from cabbage (*Brassica oleracea* var. L. *capitata*) in Zhejiang province, China in 2009 and maintained on cotton (*Gossypium hirsutum* cv. Zhe-Mian 1793). The *B. tabaci* MEAM1 culture (mtCO1 GenBank accession number KM507785) used for quantitative reverse transcription polymerase chain reaction (qRT-PCR) validation of the RNA-Seq analysis was obtained from poinsettia (*Euphorbia pulcherrima* Willd. Ex Klotzsch) in Ithaca, NY in 1989 and maintained on dwarf cherry tomato (*Solanum lycopersicum* cv. Florida Lanai). Both of these cultures were maintained in climate-controlled chambers at 27 ± 1 °C with 14 h light:10 h dark regime. The genome sequence obtained from whitefly *B. tabaci* MEAM1 maintained on collard (*Br. oleracea* ssp. acephala de Condolle) at the USDA-ARS, US Vegetable Laboratory, Charleston, SC was used to verify horizontally transferred genes (HTGs) found in RNA-Seq.

### Metabolic Reconstruction of *Portiera* and *Hamiltonella*

The metabolism genes in five published *Portiera* genomes of whitefly (NCBI: NC_018507.1, NC_018618.1, NC_018676.1, NC_018677.1, and NC_020831.1) and one *Hamiltonella* genome of the whitefly *B. tabaci* MEAM1 (European Nucleotide Archive: PRJEB7127) (Rollat-Farnier et al. 2015) were collated. Candidate metabolic pathways of *Portiera* and *Hamiltonella* were deduced using KEGG database (r63.0), EcoCyc database, and published analyses in *Buchnera* and *Hamiltonella* in aphids as guides (Shigenobu et al. 2000; Pérez-Brocal et al. 2006; Degnan et al. 2009).

### RNA Preparation and Illumina Sequencing

RNA of the whole-body whiteflies was isolated from approximately 1,000 adult female insects, using the SV total RNA isolation system (Promega) according to the manufacturer’s protocol. Because the whitefly is very small (approximately 1 mm in length) and each bacteriocyte is tiny (approximately 30 µm in diameter) and fragile, dissection of bacteriocytes is extremely time-consuming, making preparation of more than one bacteriocyte sample infeasible. Validation by qRT-PCR experiments with multiple biological replicates (primers provided in supplementary table S1, Supplementary Material online) demonstrated that one sample of pooled bacteriocytes for RNA-Seq provides an accurate measure of differential expression analyses, as previously found for mealybug bacteriocytes (Husnik et al. 2013). In total, approximately 20,000 bacteriocytes were dissected from approximately 3,000 female adult whiteﬂies using fine pins and a dissecting microscope, and RNA extractions were performed with Absolutely RNA Nanoprep Kit (Agilent) according to the manufacturer’s instructions. Each RNA sample was run on the Agilent 2100 Bioanalyzer to verify RNA quality (RIN > 6.0). RNA samples were submitted to polyA+ mRNA enrichment using oligo (dT) magnetic beads and library preparation by TruSeq RNA Sample Preparation Kit; and 90-bp paired-end libraries were sequenced by Illumina HiSeq 2000 in Beijing Genome Institute (Shenzhen, China). The raw reads are available at the National Center for Biotechnology Information (NCBI) Short Read Archive (SRA) with the accession numbers: SRR1523521 and SRR1523522, and the assembled sequences have been deposited in the NCBI’s Transcriptome Shotgun Assembly database under the accession numbers of GBII00000000 and GBIJ00000000.

### RNA-Seq and Differential Expression Analyses

Raw reads were filtered to remove low-quality reads and adaptor sequences. De novo transcriptome assemblies were carried out by the Trinity r2013-02-25 package with default settings (Grabherr et al. 2011). Each assembled transcript was searched against the NCBI nonredundant (nr) database (r20130408) and KEGG database (r63.0) using Basic Local Alignment Search Tool (BLAST) v2.2.26+x64-linux with a maximum *E* value of 1.0E^−^^5^. The insect biosynthesis pathways of amino acids, vitamins, terpenoid backbone, carotenoids and lipids as well as nitrogen metabolism were reconstructed manually using the KEGG database (r63.0). To identify differentially expressed genes between bacteriocytes and whole body, MegaBLAST was used to identify orthologous gene pairs with sequence identity greater than 99% and minimum overlapping region ≥200 bp from bacteriocyte and whole-body transcriptomes. The overlapping regions of the gene pairs were clipped out, the clean reads from the two transcriptomes were mapped, and the FPKM (fragments per kilobase of transcript per million fragments mapped) value was calculated (Mortazavi et al. 2008; Trapnell et al. 2010). Statistical comparison between two samples was performed with a custom script using the algorithm of Audic and Claverie (1997), with FDR < 0.001 and the absolute value of log_2_ ratio ≥ 1 as criteria for significantly different gene expression.

### Identification of Genes with Bacterial Origin

To identify HTGs, we adopted the pipelines used previously (Husnik et al. 2013; Sloan et al. 2014; Crisp et al. 2015) with slight modification. First, all the transcripts of bacteriocytes were subjected to BLASTX (*E* value < 1.0e^−^^10^) searches against nr database of prokaryotes, and then of eukaryotes, in NCBI. The score value and *E* value were compared between two searches for individual sequences. The HGT index, *h*, was calculated by subtracting the bitscore of the best eukaryote match from that of the best prokaryote match (Boschetti et al. 2012). Transcripts were selected for further analysis by the criteria of *h* ≥ 30, best prokaryote bitscore ≥ 100, and ln (ratio of prokaryotic:eukaryotic *E* value) < −9 in BLASTX searches against prokaryotic database; the HTGs *bioA* and *bioB* were also included, despite *h* < 30 because HTGs with these functions have previously been described in the mealybug *P. citri* (Husnik et al. 2013). The bacteriocyte transcriptome was then filtered for transcripts with FPKM > 1, to exclude possible low abundance bacterial contaminants and low-quality transcripts; transcripts with hits to *Portiera* and *Hamiltonella* were also removed. Subsequently, the BLAST hits from each transcript were individually inspected to remove likely contaminants from common environmental bacteria (e.g., *Lachnospiraceae*, *Wallemia sebi*, *Sorangium cellulosum*, etc.). Then, the candidate HTGs were checked by BLASTX manually. Transcripts with top BLASTX hit to the domain Bacteria (identity > 40) were filtered and used for the further analyses. The expression of HTG candidates between bacteriocytes and whole body was analyzed by both RNA-Seq and qRT-PCR. Finally, to check whether these HTG candidates are localized to the *B. tabaci* genome, all HTG candidates were subjected to BLASTN searches against the *B. tabaci* genome assembly (http://www.whiteflygenomics.org/). Adjacent genes on the genome scaffold were identified by BLASTX searches against nr database or BLASTN searches against nucleotide collection (nt) database. We further tested the presence of these HTGs in published transcriptome data sets of three other whitefly species: *B. tabaci* MED, *B. tabaci* Asia II 3, and *T. vaporariorum* (NCBI SRA with the accession numbers: SRX018661, SRR062575, and SRA024353.1) (Wang et al. 2010, 2012; Karatolos et al. 2011). HTG gene structure was analyzed by GENSCAN (http://genes.mit.edu/GENSCAN.html). Conserved domain of predicted proteins was analyzed using CD-search of NCBI. Amino acid alignment was conducted by BioEdit v7.1.3.0.

### Phylogenetic Analysis of HTGs

HTG candidates were searched by PSI-BLAST against the nr database. Representatives for thorough taxon-sampling were then downloaded for each HTG candidate according to its taxonomic position. Protein sequences were aligned by MAFFT V7 L-INS-i algorithm (Katoh and Toh 2008), trimmed by trimAL v1.3 with the -automated1 ﬂag set for likelihood-based phylogenetic methods, and manually corrected in BioEdit v7.1.3.0. Bayesian inference (BI) phylogenetic methods were applied to conduct the phylogenetic analyses. BI phylogenetic method used the best-fit model identified by ProtTest v2.4, and the BI analysis was conducted in MrBayes 3.2. The WAG+G+I model was used except for genes *DUR1,2* and *dapB* which required the WAG+G+I+F and RtREV+G+I+F models, respectively. In total, 10,000–55,000 trees were obtained (ngen 1,000,000–5,500,000, samplefreq 100), and the first 2,500–13,750 trees were considered as “burn in” and discarded. The potential scale reduction factor was confirmed at about 1.00 for all parameters and the average standard deviation of split frequencies was around zero. A posterior probability of each node was used for the support value of the node. Finally, phylogenetic trees were rooted by outgroups and graphically visualized in FigTree v1.4.0. Maximum-likelihood trees estimated using the program MEGA5.2 yielded very similar topologies to the Bayesian inference (BI) trees (data not shown).

### qRT-PCR Analysis

To confirm the results of RNA-Seq analyses, the expression of 27 whitefly genes, including ten HTG candidates, was measured by qRT-PCR with three biological RNA samples of isolated bacteriocytes and whole-body samples of adult whiteflies. RNA was extracted from three samples of isolated bacteriocytes and whole-body samples of adult whiteflies, as described above. The qRT-PCR primers (supplementary table S1, Supplementary Material online) were designed with Primer Premier 5.0 software (Premier Biosoft International, Palo Alto, CA). qRT-PCRs were performed using the CFX96 Real-Time PCR Detection System (Bio-Rad) with SYBR-Green detection (iQ SYBER^@^ Green Supermix; Bio-Rad). The thermal cycling protocol was as follows: Initial denaturation for 3 min at 95 °C followed by 40 cycles of 15 s at 95 °C and 30 s at 60 °C, with a melting curve analysis which confirmed that only the specific products were amplified. A 5-fold serial dilution series of whitefly cDNA was used to construct the standard curves. Each standard curve was generated by a linear regression of the plotted points. The amplification efficiency (E) of most primer pairs was in the range of 0.95–1.05, as estimated using the slope of the regression line, according to the equation: E = 10^(−^^1/slope)^ − 1. The relative expression was calculated using the 2^−^^ΔΔCt^ method and further log_2_-transformed (Livak and Schmittgen 2001) with ribosomal proteins RPL7 and RPL13 for transcript normalization.

qRT-PCR was also applied to quantify the expression of selected *Portiera* genes (supplementary table S1, Supplementary Material online), with three biological RNA samples of whole-body samples of adult whiteflies. To compare transcript abundance among genes within a metabolic pathway, the relative expression was calculated using the 2^−^^ΔCt^ method (Schmittgen and Livak 2008). Statistical significance was evaluated using one-way analysis of variance (ANOVA) at a 0.05 level in STATISTICA 6.1 (StatSoft, Inc., Tulsa, OK).

## Results

### Metabolism Genes of the Bacteriocyte Symbionts in the Whitefly *B. tabaci* MEAM1

Inspection of the four recently sequenced genomes of the primary symbiont *Portiera* in the whitefly *B. tabaci* (*Portiera-*BT) (Santos-Garcia et al. 2012; Sloan and Moran 2012; Jiang et al. 2013) revealed 99 metabolism genes, of which 50% (50 genes) contribute to the synthesis of the ten EAAs (supplementary database S1*A* and table S2*A*, Supplementary Material online). All the *Portiera-*BT genomes lack 12 genes in EAA biosynthesis, specifically in the synthesis of histidine (*hisD*), lysine (*dapF*, *lysA*) and arginine (*argABCE*) (supplementary table S2*B*, Supplementary Material online), and have two pseudogenes (*argH* in arginine synthesis, and *dapB* in lysine synthesis) and one further gene, *argG*, likely undergoing pseudogene formation (supplementary fig. S1*A–C*, Supplementary Material online, with criteria for pseudogene designations in legend). Furthermore, the total metabolic functions of *Portiera*, as predicted from its gene content, require eight classes of cofactors (thiamine, nicotinamide, pyridoxal 5′ phosphate, folic acid, FMN/FADH, ubiquinone-n, heme: supplementary table S2*C*, Supplementary Material online), none of which can be synthesized by this bacterium.

The metabolism genes in the sequenced genome of *Hamiltonella* from *B. tabaci* MEAM1 (Rollat-Farnier et al. 2015) were then analyzed to investigate whether *Hamiltonella*, which co-occurs with *Portiera* in every bacteriocyte of *B. tabaci* MEAM1, compensates for the missing genes in *Portiera*. Although *Hamiltonella* lacks most genes in EAA biosynthesis, it does have *dapB*, *dapF* and *lysA*, the three genes in lysine biosynthesis that are absent or pseudogenized in *Portiera*, raising the possibility that the metabolic pathway for lysine synthesis may be shared between *Portiera* and *Hamiltonella*. *Hamiltonella* also has the complete biosynthetic pathways for six of the eight cofactors that are required for its own metabolism and by *Portiera* (supplementary table S2*C*, Supplementary Material online), but lacks the entire thiamine biosynthetic pathway and one gene in folate biosynthesis, alkaline phosphatase (*phoAB*).

### The Transcriptome of the *B. tabaci* Bacteriocyte

To investigate the role of host genes compensating for missing *Portiera* metabolism genes, the transcriptome of dissected bacteriocytes of *B. tabaci* MEAM1 was quantified by RNA-Seq, and then interrogated for genes for coding enzymes missing from EAA biosynthesis and other metabolic pathways in *Portiera*. The RNA-Seq libraries derived from bacteriocytes and the whole body of whiteflies yielded 52.4 and 51.8 million reads, respectively (supplementary table S3, Supplementary Material online). In total, 7,900 transcripts were differentially expressed between bacteriocytes and the whole body of whiteflies with FDR < 0.001 (supplementary database S1*B*, Supplementary Material online), of which 4,752 (60%) were enriched and 3,148 were depleted in the bacteriocytes. qRT-PCR analysis of the expression of 27 genes using two reference genes verified the reliability of the RNA-Seq analysis (supplementary table S4*A*, Supplementary Material online).

### Metabolism Genes of Bacterial Origin in the Host Genome

Many of the missing *Portiera* genes in EAA and cofactor synthesis code for reactions that cannot generally be mediated by animals. Therefore, we interrogated the transcriptome for candidate genes of bacterial origin. Following the exclusion of sequences assigned to the symbionts *Portiera* and *Hamiltonella,* ten metabolism genes of bacterial origin were recovered (table 1 and supplementary table S5*A*, Supplementary Material online): Six involved in EAA synthesis: arginine (*argG* and *argH*), lysine (*dapB*, *dapF*, and *lysA*), and phenylalanine (*CM*); two mediating urea degradation: *DUR1,2* coding both reactions (urea carboxylase and allophanate hydrolase) and *AH* coding the allophanate hydrolase reaction only; and two in the synthesis of the cofactor biotin (*bioA* and *bioB*). BLAST searches against the genome assembly of *B. tabaci* MEAM1 confirmed that each of the ten gene sequences was located on genome scaffolds of substantial size (11.2 kb–12.7 Mb; supplementary table S5*A*, Supplementary Material online) and flanked by bona fide insect genes (table 1 and supplementary table S5*B*, Supplementary Material online). Five of the ten genes have eukaryotic GT–AG introns (supplementary fig. S2, Supplementary Material online). The expression of all the HTGs was ≥1.5-fold greater in bacteriocytes than the whole body, as quantified by both RNA-Seq and qRT-PCR, apart from *bioB* which differed by ≤20% between the bacteriocytes and whole body (table 1 and supplementary table S4*A*, Supplementary Material online).

**Table 1:** The Expressed HTGs Found in *Bemisia tabaci* whiteflies

Gene Name	Description	Bacteriocyte Expression	Whole-Body Expression	Fold Change (Log_2_ Ratio)		Intron	Phylogenetic Origin
		(FPKM)	(FPKM)				
				**RNA-Seq**	**qRT-PCR****^a^**		
*argG*	Argininosuccinate synthase [EC:6.3.4.5]	407.68	236.06	0.79	3.44	+	Gammaproteobacteria: Enterobacteriales
*argH*	Argininosuccinate lyase [EC:4.3.2.1]	73.05	27.36	1.42	4.4	+	Gammaproteobacteria: Enterobacteriales
*dapB*	4-Hydroxy-tetrahydrodipicolinate reductase [EC:1.17.1.8]	567.70	40.64	3.80	4.78	−	Alphaproteobacteria: Rickettsiales
*dapF*	Diaminopimelate epimerase [EC:5.1.1.7]	13.67	7.13	0.94	5.75	+	Gammaproteobacteria: Enterobacteriales
*lysA*	Diaminopimelate decarboxylase [EC:4.1.1.20]	311.44	22.28	3.81	5.31	−	Planctomycetes
*CM*	Chorismate mutase [EC:5.4.99.5]	242.25	123.56	0.97	2.74	+	Gammaproteobacteria: Enterobacteriales
*bioA*	Adenosylmethionine-8-amino-7-oxononanoate aminotransferase [EC:2.6.1.62]	8.85	5.36	0.72	1.72	−	Alphaproteobacteria or Bacteroidetes or Betaproteobacteria
*bioB*	Biotin synthase [EC:2.8.1.6]	4.61	4.26	0.12	0.27	+	Alphaproteobacteria: Rickettsiales
*AH*	Allophanate hydrolase [EC:3.5.1.54]	14.96	2.05	2.87	5.05	−	Bacteroidetes
*DUR1,2*	Urea carboxylase/allophanate hydrolase [EC:6.3.4.6 EC:3.5.1.54]	4.23	2.44	0.80	3.46	−	Gammaproteobacteria: Enterobacteriales

Note.—“+” indicates presence, “−” indicates absence.

^a^The fold-difference in expression between bacteriocytes and whole body (log_2_) scale, relative to the normalizing gene RP7 is displayed (mean ± SE, three biological replicates). For all the data using two normalizing genes (RP7 and RP13), see supplementary table S4*A*, Supplementary Material online.

We additionally tested published whitefly transcriptome data sets (Wang et al. 2010, 2012; Karatolos et al. 2011) for the presence of these HTGs. Seven of the ten genes (all except *argH*, *DUR1,2*, and *AH*) were detected in the transcriptome of *B. tabaci* Asia II 3 (also known as ZHJ1 biotype); eight genes (all except *DUR1,2* and *AH*) were detected in the transcriptome of *B. tabaci* MED (supplementary fig. S2*H* and table S5*C*, Supplementary Material online); and three genes (*CM*, *bioA*, and *bioB*) were detected in the transcriptome of *T. vaporariorum* (supplementary table S5*C*, Supplementary Material online). These data indicate that at least three gene transfer events (*CM*, *bioA*, and *bioB*) are relatively ancient, with at least seven events present in multiple species of the *B. tabaci* complex.

Having established the localization of the ten HTGs of bacterial origin to the *B. tabaci* genome and their expression in the bacteriocytes, we investigated their phylogenetic origins. Although genes with identical annotations to all of the candidate HTGs except *AH* and *DUR1,2* are borne by one or both of *Portiera* and *Hamiltonella*, the amino acid sequence identity between each candidate HTG and the symbiont homolog(s) was less than 50% (supplementary table S5*D*, Supplementary Material online), indicating that the whitefly genome assembly was not contaminated by whitefly symbiont sequences. The identification of seven HTGs in the transcriptome of *B. tabaci* Asia II-3 (supplementary fig. S2*H*, Supplementary Material online), which does not bear *Hamiltonella*, provides further evidence against *Hamiltonella* as the source of these genes.

Phylogenetic analysis assigned the HTGs to diverse bacterial lineages. Five HTGs (*dapF*, *argG*, *argH*, *CM*, and *DUR1*) cluster within Enterobacteriales (γ-proteobacteria) (table 1 and supplementary fig. S3, Supplementary Material online), including four that group with *Pantoea* and gut symbionts in stinkbugs (*dapF* and *argH* in bacterial symbionts of *Halyomorpha halys* and *Plautia stali*, respectively). *DapB* and *bioB* cluster with the Rickettsiales (α-proteobacteria), *AH* with *Niastella* (Bacteroidetes), and *lysA* with *Isosphaera* (Planctomycetes). Exceptionally, the phylogenetic position of *bioA* clusters with three divergent bacterial taxa, Rickettsiales (α-proteobacteria), *Cardinium* (Bacteroidetes) and β-proteobacteria, perhaps indicative of horizontal transfer of this gene across these bacterial phyla.

### Complementarity and Duplication between the Metabolism Gene Content of the Host Bacteriocyte and *Portiera*

**Fig. 1.—:**
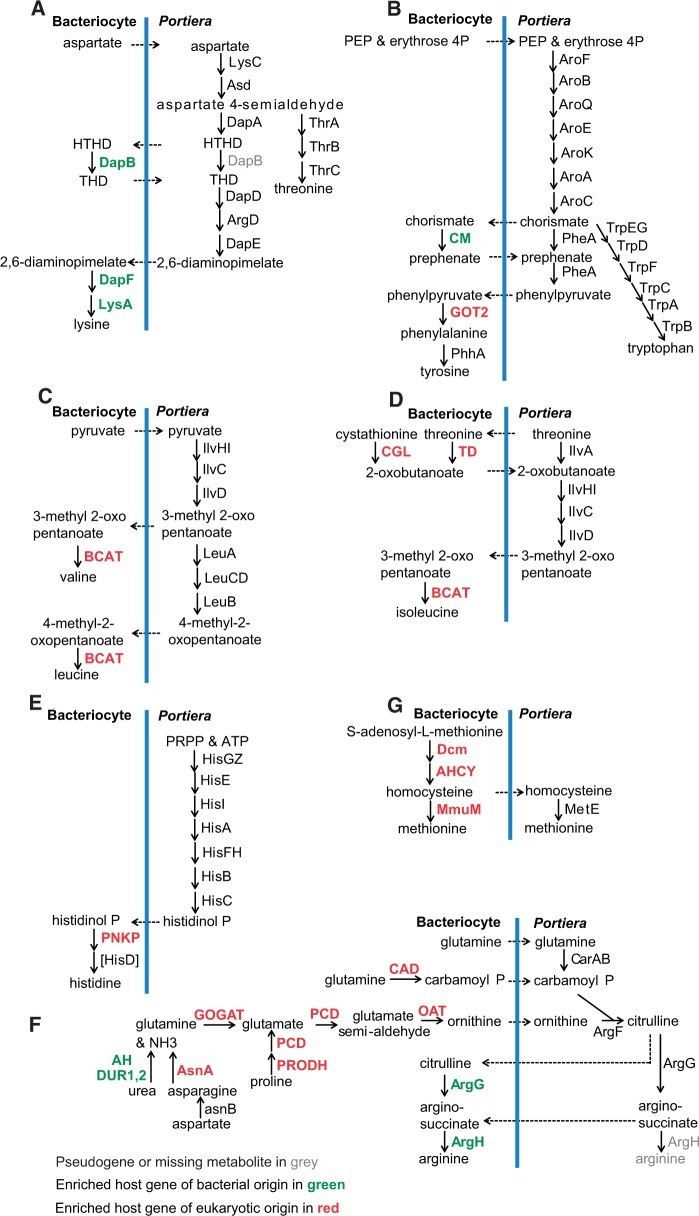
Cooperation of EAA synthesis between whiteflies and *Portiera. Portiera* pseudogenes are indicated in gray and host genes with enriched transcript abundance in the bacteriocytes (RNA-Seq data) are indicated in red (genes of insect origin) or green (genes of bacterial origin). (*A*) Lysine and threonine synthesis (HTHD, (2S,4S)-4-hydroxy-2,3,4,5-tetrahydrodipicolinate; THD, 2,3,4,5-tetrahydrodipicolinate). (*B*) Phenylalanine and tryptophan synthesis. (*C*) Valine and leucine synthesis. (*D*) Isoleucine synthesis. (*E*) Histidine synthesis (PNKP). (*F*) Arginine synthesis (AH, allophanate hydrolase; Dur1,2, urea carboxylase/allophanate hydrolase; AsnA, asparaginase; GOGAT, glutamate synthetase; PCD, delta-1-pyrroline-5-carboxylate dehydrogenase; PRODH, proline dehydrogenase). (*G*) Methionine synthesis; the insect can synthesize methionine as part of the *S*-adenosyl methionine cycle involving the enzymes DNA-(cytosine-5) methyl transferase (Dcm), adenosyl homocysteinase (AHCY), and homocysteine 5-methyl transferase (MMUM).

Analysis of the bacteriocyte transcriptome identified nine host genes that mediate reactions missing from the EAA biosynthesis gene complement of *Portiera*. Two of the genes are HTGs: *dapF* and *lysA*, which complement the missing genes coding the two terminal reactions in lysine synthesis (fig. 1*A*, table 1). The genes of intrinsic (i.e., insect) origin are *GOT2*, BCA-transferase (*BCAT*), and polynucleotide kinase 3′-phosphatase (*PNKP*), which is a bifunctional polynucleotide phosphatase/kinase containing the *hisB* domain (fig. 1*B*–*E*), compensating, respectively, for *tyrB, ilvE* and *hisB* missing from *Portiera*, and the insect genes synthesizing ornithine (an intermediate in arginine synthesis) from glutamate/glutamine, which replace the bacterial *argABCE* missing from the *Portiera* genome (fig. 1*F*). In addition, the insect apparently lacks the reactions coded by the missing *Portiera* genes *metABC*, which transform homoserine to homocysteine, the substrate for MetE-mediated methionine synthesis by *Portiera* (although the host can synthesize homocysteine by DNA-(cytosine-5) methyl transferase and adenosyl homocysteinase, with *S*-adenosyl methionine as substrate) (fig. 1*G*).

We identified several instances of apparent metabolic duplication between the EAA biosynthesis genes in the bacteriocyte and *Portiera* (fig. 1 and supplementary database S1*C*, Supplementary Material online). These include the HTGs *argG*, *argH*, *dapB*, and *CM* (fig. 1*A*, *B*, and *F*) despite *Portiera* bearing equivalent genes (*argG*, *argH*, *dapB*, and *pheA*, respectively); the insect genes threonine dehydratase (*TD*) and cystathionine-γ-lyase (*CGL*), which yield the same reaction product (2-oxobutanoate) as the *Portiera ilvA* (fig. 1*D*); the insect genes carbamoyl-phosphate synthase/aspartate carbamoyltransferase/dihydroorotase (*CAD*) providing an alternative reaction to the *Portiera* carbamoyl-phosphate synthase (*carAB*) for the synthesis of carbamoyl-P, a key substrate in arginine synthesis (fig. 1*F*); and homocysteine-*S*-methyltransferase, mediating the transformation of homocysteine to methionine, in parallel with the *Portiera metE* (fig. 1*G*).

**Fig. 2.—:**
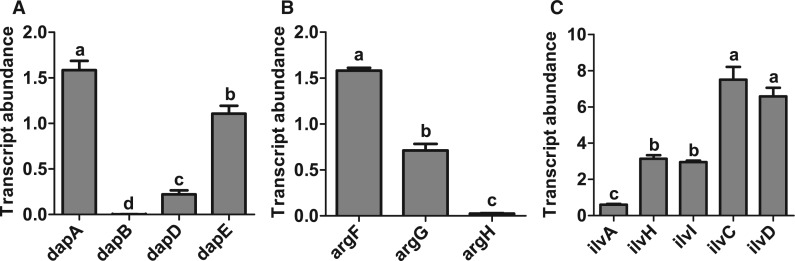
Transcript abundance of lysine (*A*), arginine (*B*), and isoleucine (*C*) synthesis pathway genes in *Portiera*. The transcript abundance (2^−ΔCt^) of *Portiera* genes, relative to the normalizing gene RP7 is displayed (mean ± SE, three biological replicates). Different letters indicate significant difference. Data for a second normalizing gene, RP13, yielded similar results (see supplementary table S4*B*, Supplementary Material online).

Among the equivalent *Portiera* genes, *argH* and *dapB* are pseudogenes and *argG* is likely evolving to a pseudogene (supplementary fig. S1*A–C*, Supplementary Material online), but the *ilvA* and *pheA* of *Portiera* are apparently intact (supplementary fig. S1*D* and *E*, Supplementary Material online). Furthermore, the expression of the *Portiera dapB*, *ilvA, argG*, and *argH* genes is significantly reduced compared with other genes in the same metabolic pathways (fig. 2 and supplementary table S4*B*, Supplementary Material online). Although flux through a reaction cannot be inferred precisely from the sequence or abundance of the gene transcript because of possible posttranscriptional controls (Hansen and Degnan 2014; Russell et al. 2014), these data suggest that, where host and symbiont bear genes with equivalent metabolic function in EAA synthesis, the functional integrity or expression of the symbiont gene is compromised.

**Fig. 3.—:**
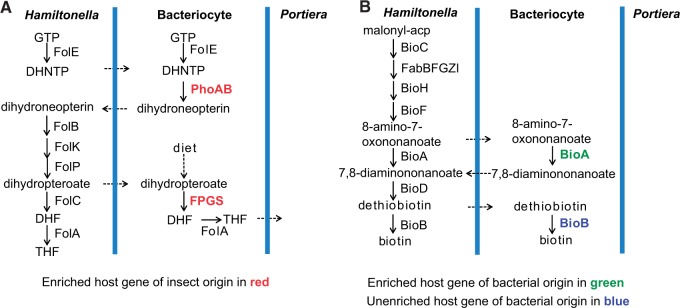
Cooperation of cofactor synthesis between whiteflies and *Hamiltonella*. (*A*) Folate synthesis. DHNTP, 7,8-dihydroneopterin 3′-triphosphate. (*B*) Biotin synthesis.

Genetic complementarity and duplication are also evident between the bacteriocyte and *Hamiltonella*, in relation to cofactor biosynthesis. The bacteriocyte transcriptome is enriched in *phoAB*, the single gene in folate biosynthesis missing from the *Hamiltonella* genome, suggesting that folate synthesis from GTP may be shared between *Hamiltonella* and the host (fig. 3*A*). Duplication is illustrated by the biotin synthesis pathway, which is complete in the *Hamiltonella* genome and is defined by the HTGs *bioA* and *bioB* in the host genome (fig. 3*B*). The evidence that both HTGs are expressed in bacteriocytes, including enriched expression of *bioA* (table 1 and supplementary table S5*A*, Supplementary Material online), suggests that the bacteriocyte contributes to the flux through these two reactions, with the net transfer of up to three metabolic intermediates between the *Hamiltonella* cells and the bacteriocyte (fig. 3*B*). Although the cofactor biotin is apparently not required by *Portiera* (supplementary table S2*C*, Supplementary Material online), it is needed for carboxylation reactions in host fatty acid biosynthesis and other pathways (e.g., TCA cycle) that generate metabolites required by *Portiera* (see below). The bacteriocyte is also enriched in transcripts for many of the enzymes that process cofactors (supplementary fig. S4*A–I*, Supplementary Material online), indicating that the host is metabolically poised to provide *Portiera* with cofactors that may be derived from the diet or *Hamiltonella*.

The bacteriocytes also have elevated transcript abundance of metabolism genes contributing to predicted precursors of *Portiera* metabolism, including ornithine (for arginine synthesis), PRPP (precursor of histidine synthesis), coenzyme A (in leucine and lysine synthesis), and geranylgeranyl diphosphate (for carotenoid synthesis) (fig. 1*F*, supplementary fig. S4*J*–L and database S1*C*, Supplementary Material online). Transcripts of genes in fatty acid synthesis and desaturation, and glycerolipid, phospholipid and sphingolipid synthesis are also enriched in bacteriocytes (supplementary fig. S4*M*, Supplementary Material online). This enhanced metabolic capability is predicted to contribute to the lipid requirement of *Portiera*, which has no capacity for lipid biogenesis, and the synthesis of the symbiosome membrane of insect origin that bounds each bacterial cell within the bacteriocyte. Consistent with this interpretation, multiple PE-binding proteins and phospholipid-transporting ATPase are enriched in bacteriocytes, indicative of intense phospholipid transportation activity (supplementary database S1*C*, Supplementary Material online).

### Comparative Analysis of Host–Symbiont Metabolism Genes in Sternorrhynchan Insects

Genome-based studies of host–symbiont metabolic interactions have been published for three of the four superfamilies of sap-feeding sternorrhynchan hemipteran insects (Hansen and Moran 2011; Poliakov et al. 2011; Macdonald et al. 2012; Husnik et al. 2013; Sloan et al. 2014). Our investigation of the whitefly *B. tabaci*, a member of the remaining superfamily (Aleyrodoidea), provides the opportunity for comparisons among the patterns of metabolic coevolution in these systems.

The bacteriocytes in all four systems have abundant or elevated expression of orthologous host genes of intrinsic origin that code for reactions missing from their symbionts (table 2). These are *GOT*, with function matching the bacterial *tyrB*, missing from all symbionts; *BCAT*, matching *ilvE*, absent from the symbionts in all insects except the psyllid; one or both of *TD* and *CGL*, which generate 2-oxobutanoate, the product of the reaction coded by the bacterial gene *ilvA*, missing from all the bacterial symbionts except *Portiera*; and *OAT*, which produces ornithine, an intermediate in arginine synthesis and product of the bacterial *arg* genes that either are missing (three symbioses) or have reduced expression (the aphid-*Buchnera* symbiosis). Multiple reactions are known to generate the substrate of OAT, 1-pyrroline-5-carboxylate, and current data implicate different enzymes in the different symbioses (table 2).

**Table 2:** Relationship between Symbiont Gene Content and Bacteriocyte Expression of Host Gene(S) Coding the Equivalent Reactions

Symbiosis	Amino Acids										
	Branched Chain Amino Acids^a^		Phenylalanine^b^		Isoleucine^c^			Arginine^d^			
	Symbiont	Host	Symbiont	Host	Symbiont	Host	Host	Symbiont	Host	Host	Host
	*ilvE*	*BCAT*	*tyrB*	*GOT*	*ilvA*	*TD*	*CGL*	*argABCDE*	*PCD*	*PRODH*	*OAT*
Whitefly (*Bemisia tabaci/Portiera*)	0	+	0	+	+	+	+	0	+	+	+
Mealybug (*Planococcus citri/Tremblaya+Moranella*)	0	+	0	+	0		+	0	?	?	+
Psyllid (*Pachypsylla venusta/Carsonella*)	+	?	0	+	0	+	+	0			+
Aphid (*Acyrthosiphon pisum/Buchnera*)	0	+	0	+	0		+	+		+	+

Note.—Symbiont: gene present (+) or absent (0); host: abundant or enriched transcript in bacteriocytes (+); blank cell, no information.

^a^The final reaction in the synthesis of branched chain amino acids mediated by IlvE or BCAT (branched chain amino acid transaminase, which generally functions as the first reaction in BCA degradation but is reversible).

^b^The final reaction in phenylalanine synthesis mediated by the bacterial TyrB and also by host transaminases, notably aspartate transaminase (GOT, glutamate-oxaloacetate transaminase).

^c^The first reaction in the isoleucine synthesis, producing 2-oxobutanoate from the substrate threonine (bacterial IlvA and host TD) or homoserine (host CGL). IlvN, coding the second reaction in isoleucine synthesis, is also missing from the symbionts of the mealybug symbiosis, and it is unknown how this reaction may be mediated.

^d^Synthesis of ornithine, an intermediate in arginine synthesis, from glutamate by the bacterial argABCDE or host PCD (1-pyrroline-5-carboxylate dehydrogenase) and OAT (ornithine aminotransferase), or from proline by the host PRODH (proline dehydrogenase), PCD and OAT.

**Fig. 4.—:**
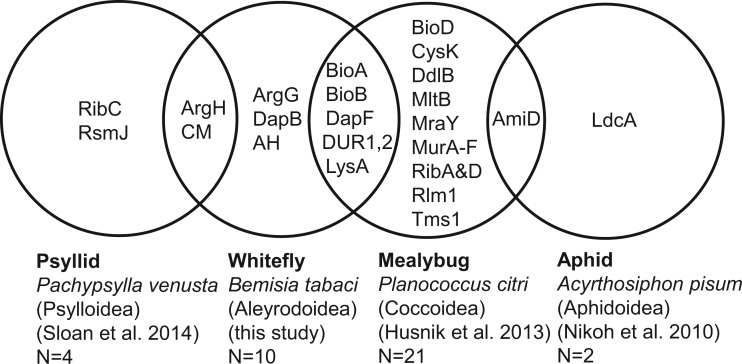
Comparison of HTGs in whiteflies and other sternorrhynchan insects. All functions shared by different insects are represented by genes from different bacterial taxa (e.g., ArgH from Enterobacteriales in the whitefly and Halomonadaceae in the psyllid), with the possible exception of BioB (of α-proteobacterial origin) shared between the whitefly and mealybug.

Genes with the same functional annotation as seven of the ten HTGs of *B. tabaci* MEAM1 (i.e., all but *AH*, *argG*, and *dapB*) have previously been identified as HTGs in the genomes of the psyllid *Pachypsylla venusta* and the mealybug *P. citri* (Husnik et al. 2013; Sloan et al. 2014), but not in the aphid *Acyrthosiphon pisum* (fig. 4). Both the divergent phylogenetic position and low amino acid sequence identity between each pair of functionally equivalent genes (supplementary fig. S3, Supplementary Material online) indicate independent transfers of *argH* and *CM* between *B. tabaci* and the psyllid, and *DUR1,2*, *lysA* and *dapF* between *B. tabaci* and the mealybug *P. citri*. Phylogenetic analysis of the remaining two genes yields an ambiguous tree topology for *bioA* and indicates that *bioB* may be derived from a single evolutionary acquisition that preceded the diversification of whiteflies (superfamily Aleyrodoidea) and mealybugs (members of the superfamily Coccoidea) (supplementary fig. S3, Supplementary Material online). This scenario requires the evolutionary loss of *bioB* from the lineage giving rise to the aphids (superfamily Aphidoidea), the sister group of the Coccoidea. The size and position of introns in *bioB* in the whitefly and mealybug are very different (supplementary fig. S2*G*, Supplementary Material online vs. table S3 in Husnik et al. [2013]), indicating that the introns were acquired independently.

## Discussion

Evolutionary change in obligately vertically transmitted bacteria is dominated by genomic deterioration, resulting in reduced gene function and gene loss (Moran 1996; Moran and Bennett 2014). These intracellular bacteria are generally isolated from the horizontal acquisition of genetic material from bacteriophage, plasmids, and so forth, linked to the decay of DNA recombination and repair genes, so precluding the recovery of lost function from incoming DNA. Their otherwise inexorable gene loss is countered by selection for nutritional function advantageous to the animal host. This selective barrier against gene loss can, however, be circumvented by two processes: The evolution of host functions that duplicate the function of bacterial genes (Wilson et al. 2010; Russell et al. 2013), and the acquisition of alternative symbionts that can mediate equivalent functions, resulting in the displacement of the ancestral bacterium or partitioning of function between the two bacteria (Pérez-Brocal et al. 2006; McCutcheon and Moran 2007; Koga and Moran 2014). This study on the relationship of the whitefly *B. tabaci* MEAM1 with the primary symbiont *Portiera* and secondary symbiont *Hamiltonella* offers insights into these two processes.

Our genomic and transcriptomic analyses of the *B. tabaci* have revealed multiple genes in the whitefly genome that compensate for the deterioration and loss of EAA biosynthesis genes in the primary symbiont *Portiera*, as previously demonstrated for representatives of other members of the suborder Sternorrhyncha, the psyllids, mealybugs and aphids (Wilson et al. 2010; Hansen and Moran 2011; Poliakov et al. 2011; Husnik et al. 2013; Russell et al. 2013; Sloan et al. 2014), and the compensating genes in the whitefly genome include both genes of insect origin and genes horizontally acquired from other bacteria. Three aspects of these results are of note. First, no intraspecific variation in gene content or expression patterns were identified across the multiple analyses of this study (which used three isolates, see Materials and Methods) and published sequence data (table 1), suggesting that the genetic basis of nutritional interactions in this symbiosis in *B. tabaci* MEAM1 may be relatively uniform. Second, none of the HTGs identified in *B. tabaci* and other insects, apart probably from *argH* in the psyllid *Pa. venusta* (Sloan et al. 2014), is derived from bacteriocyte symbionts, even though these bacteria are invariable residents that colonize every unfertilized egg through vertical transmission, providing repeated opportunities for DNA transfer to the gamete. Most symbiont genes have a high AT content (McCutcheon and Moran 2012) and their protein products have low predicted stability (Bastolla et al. 2004), with high concentrations of GroEL and other chaperone proteins believed to be required for function (Fares et al. 2002; Kupper et al. 2014). These traits may reduce function in the nucleocytoplasmic compartment of any gene derived from bacteriocyte symbionts. Third, despite considerable overlap in the function of the transferred genes, most of the HTGs with the same function in whiteflies, psyllids and mealybugs are derived from phylogenetically distinct bacteria and, consequently, are independent acquisitions (fig. 4). These data reinforce the conclusions from various studies that animal genomes can support functional genes of bacterial origin (Starcevic et al. 2008; Danchin et al. 2010; Altincicek et al. 2012; Ahn et al. 2014) and, furthermore, suggest that genes in EAA synthesis are of great selective advantage to hosts of EAA-producing bacteria subject to genomic decay.

Current understanding of the process by which enzymes coded by genes in the host genome contribute to bacterial metabolic pathways, such that the metabolic pathways are shared between the partners, comes from research on the aphid-*Buchnera* symbiosis. Unlike bacterial-derived organelles, metabolic enzymes coded by the host nucleus are not targeted to *Buchnera* cells (Poliakov et al. 2011) (although immunocytochemical data of Nakabachi et al. [2014] suggest that one host protein is localized to the *Buchnera* cells). Instead, the metabolic intermediates are translocated between the bacterial cells and host compartment of the bacteriocytes (Russell et al. 2013). Consequently, the shared metabolic pathways are founded on evolutionary changes in both the regulation of specific host metabolic genes to favor expression in the bacteriocytes and also transporter functions for the transfer of metabolic intermediates across the *Portiera* cell membrane and bounding symbiosome membrane. The resultant transfer of metabolic function from symbiont to host may be of selective advantage in two complementary ways, both of which would facilitate further deterioration in function of the symbiont gene. First, host function can compensate for reduced function of bacterial enzymes caused by genomic decay. Additionally, the host enzyme activity is likely to alter metabolite flux through the metabolic pathway, resulting in host control over EAA production, and so confer a selective advantage for shared metabolic pathways independent of symbiont gene decay. This may account for the several instances of host enzymes that duplicate apparently functional enzymes in the symbiont. For example, in isoleucine synthesis, the first committed reaction is mediated by the bacterial gene *ilvA*, which is structurally intact in *Portiera* but expressed at reduced levels (supplementary fig. S1*E*, Supplementary Material online, and fig. 2*C*). This condition and associated elevated bacteriocyte expression of *TD* and *CGL*, both of which yield the IlvA product, 2-oxobutanoate, are comparable to a likely intermediate stage in the evolution of the aphid symbiosis, where elevated bacteriocyte *CGL* is inferred to compensate for the missing *Buchnera ilvA* (Poliakov et al. 2011). Similarly, the replacement of *Portiera argABCDE* mediating the synthesis of ornithine (precursor of arginine) by host enzymes is presaged by the reduced expression of apparently intact *argABCDE* in *Buchnera*, associated with elevated bacteriocyte expression of host genes producing ornithine (Poliakov et al. 2011).

Horizontal gene transfer typically involves one or a few cotransferred genes and is widely recognized as a route for the acquisition of genetically simple novel traits, but not multigene functions, such as a complete metabolic pathway. However, the pattern of arginine synthesis in the whitefly reveals how symbiotic bacteria subject to genomic decay can enable the stepwise acquisition of metabolic pathways by horizontal gene transfer. Genome reconstructions indicate that the common ancestor of Hemiptera lacked the genetic capacity for arginine synthesis from ornithine. Multiple hemipterans can utilize arginine-insufficient diets by their symbiosis with bacteria that synthesize this amino acid. In the presence of this capability, sequential acquisitions of individual genes coding single reactions in arginine biosynthesis can be selected because each HTG can function in conjunction with other genes of the pathway coded by the bacterial symbiont, while compensating for (or facilitating) the decay of the bacterial gene coding for the same reaction. In principle, a host bearing bacteria subject to genomic decay can acquire the complete metabolic pathway by stepwise accumulation of genes coding individual reactions. For the whitefly symbiosis, all the *arg* genes in the ancestral *Portiera* except *argF* are also represented in the *B. tabaci* bacteriocyte, and a future host acquisition of a gene coding the ArgF reaction would render the host completely independent of the symbiosis for arginine synthesis. At that point, selection would be relaxed for enriched expression of arginine synthesis genes in the bacteriocyte relative to other insect tissues, and the whitefly lineage would recover the ancestral animal condition, in which animal genes mediate arginine synthesis de novo (Guedes et al. 2011).

Additional bacteria recruited to the symbiosis, variously known as secondary symbionts or auxiliary symbionts, have also been implicated as a source of EAA and cofactor biosynthesis genes (McCutcheon and Moran 2007; Gosalbes et al. 2008). Our genomic analysis of *Hamiltonella* suggests that this bacterium may, similarly, contribute reactions to the shared pathway for lysine synthesis in *B. tabaci* MEAM1, and also synthesize multiple cofactors that, if released, may contribute to the cofactor requirement of the insect and *Portiera*. The comparative genomic analysis of metabolic functions of independently evolved bacteria and bacteriocytes in the different sap-feeding insects reveals multiple instances of convergent evolution. This applies particularly to the selective expression of individual host genes in bacteriocytes, generally out of context of other genes contributing to host metabolic pathways, and also to the metabolism-related HTGs in the *Bemisia* genome, 70% of which are functionally equivalent to HTGs in other symbioses. Across a broader phylogenetic perspective, however, the overlap in the HTGs in the different symbioses is more limited, with no functionally equivalent HTGs present in all four sternorrhynchan groups (fig. 4). These data suggest that the functional properties of DNA fragments that happen to be acquired from transient bacterial infections may play an important role in shaping the metabolic traits of these associations. In other words, contingency may contribute to the evolutionary trajectory of these associations, and “rewinding the evolutionary tape” does not necessarily lead to the same evolutionary outcome.

In conclusion, this analysis of bacterial symbioses in plant sap-feeding insects reveals substantial evolutionary remodeling of the genomes of both insect host and bacterial symbiont, in response to the genomic deterioration of the bacterial partner, with consequences that both compensate for and facilitate further genomic decay. The key priorities for future research are to determine the molecular processes underpinning bacteriocyte-specific expression of genes in the host genome, and the genetic and metabolic mechanisms by which nutrient flux between bacteria and bacteriocyte is coordinated with the dietary supply of nutrients.

## Supplementary Material

Supplementary database S1, figures S1–S4, and tables S1–S5 are available at *Genome Biology and Evolution* online (http://www.gbe.oxfordjournals.org/).

Supplementary Data
