# Supplementary material for: Metabolic Coevolution in the Bacterial Symbiosis of Whiteflies and Related Plant Sap-Feeding Insects
Source: Genome Biol Evol. 2015 Sep 15;7(9):2635–47. doi: 10.1093/gbe/evv170 (PMC4607527; doi:10.1093/gbe/evv170)
Supplement: Supplementary Data [file supp_7_9_2635__index.html]

Metabolic coevolution in the bacterial symbiosis of whiteflies and related plant sap-feeding insects — Metabolic Coevolution in the Bacterial Symbiosis of Whiteflies and Related Plant Sap-Feeding Insects — Supplementary Data 

# Metabolic Coevolution in the Bacterial Symbiosis of Whiteflies and Related Plant Sap-Feeding Insects

## Supplementary Data

files

- Supplementary Data - zip file
