# Supplementary material for: Metabolic Coevolution in the Bacterial Symbiosis of Whiteflies and Related Plant Sap-Feeding Insects
Source: Genome Biol Evol. 2015 Sep 15;7(9):2635–47. doi: 10.1093/gbe/evv170 (PMC4607527; doi:10.1093/gbe/evv170)
Supplement: Supplementary Data [file supp_evv170_suppl_data.zip › Fig. S1.pdf]

Fig. S1

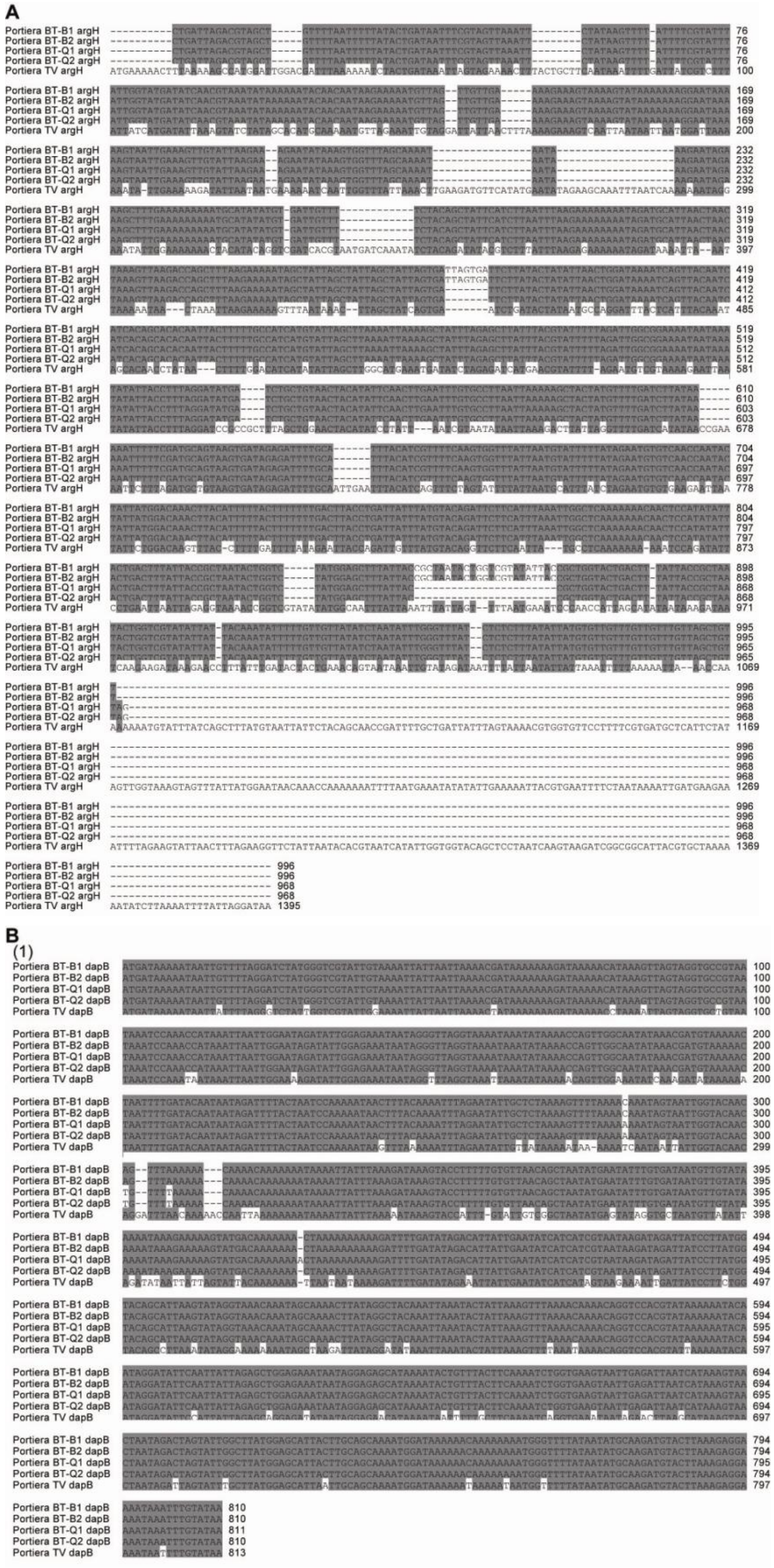
A
Portiera BT-B1 argH
Portiera BT-B2 argH
Portiera BT-Q1 argH
Portiera BT-Q2 argH
Portiera TV argH
B
(1)
Portiera BT-B1 dapB
Portiera BT-B2 dapB
Portiera BT-Q1 dapB
Portiera BT-Q2 dapB
Portiera TV dapB

(2)

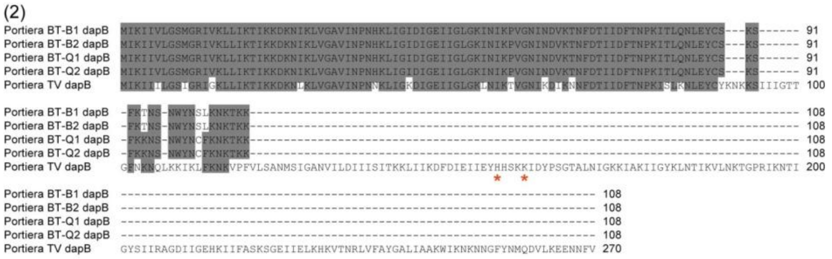

(3)

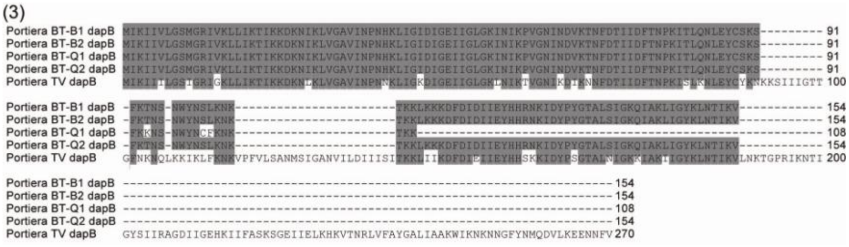

c

(1)

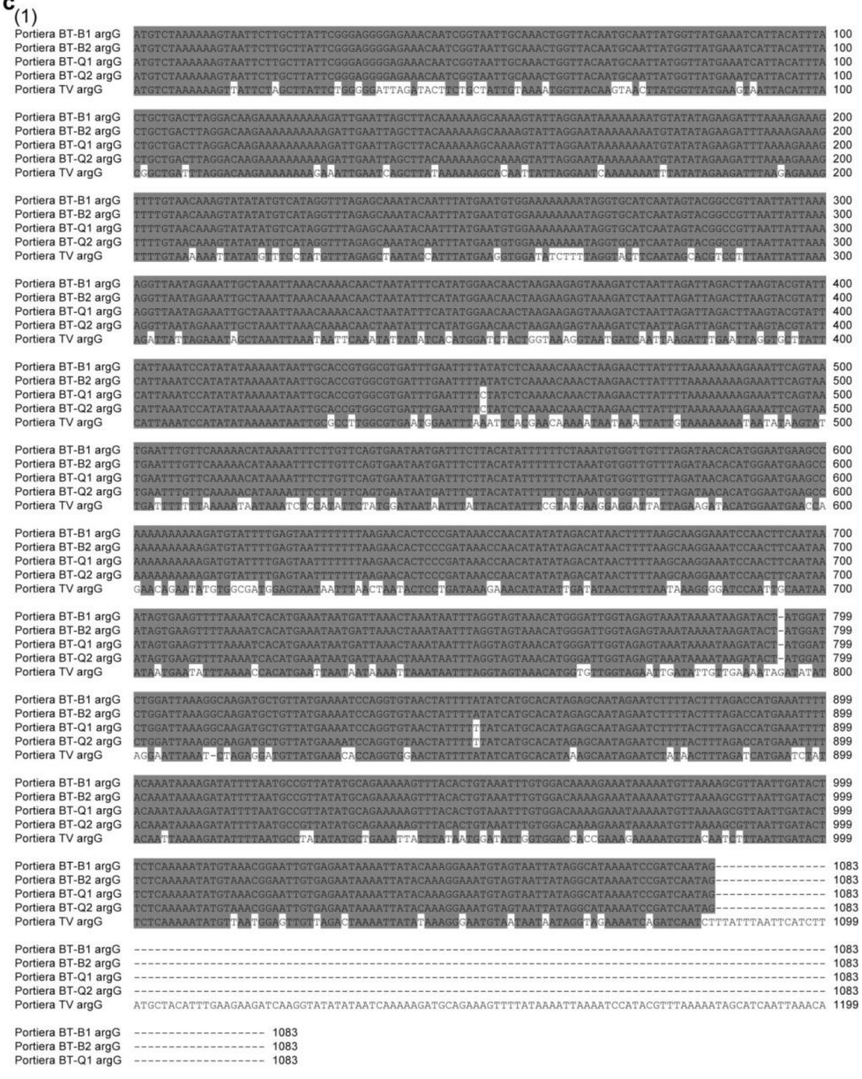

(2)

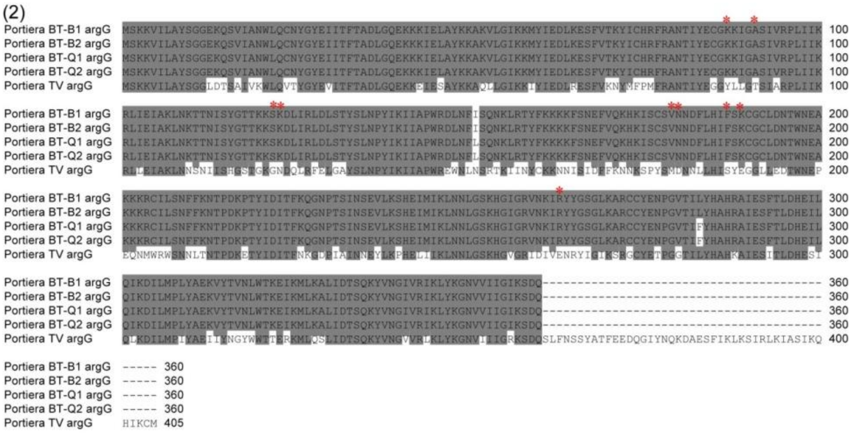

D

(1)

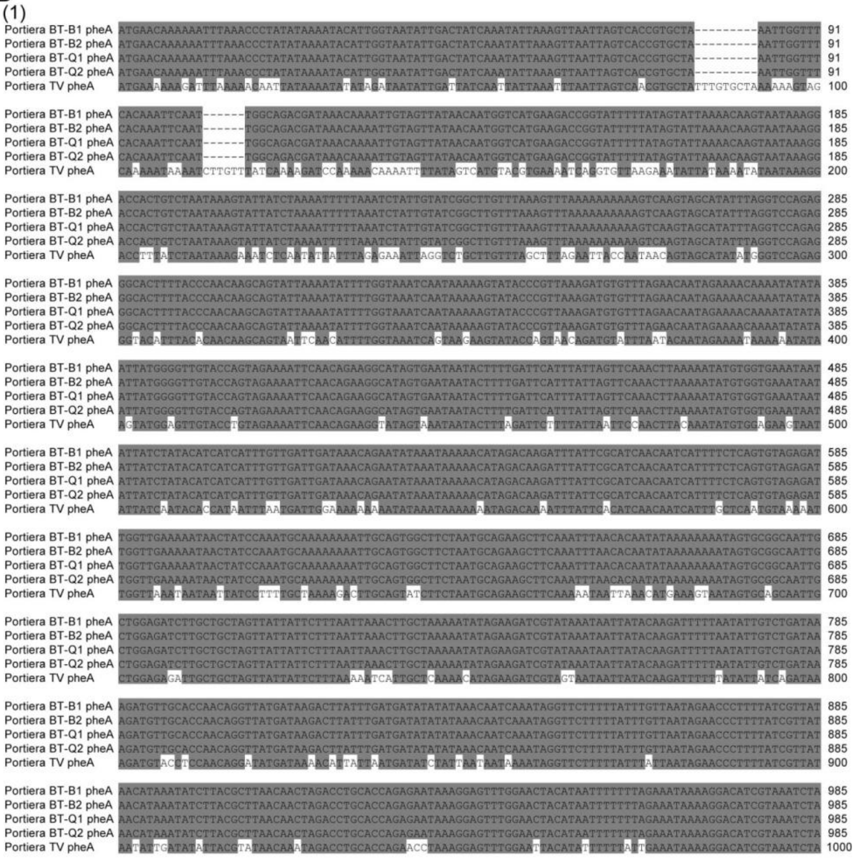

(2)

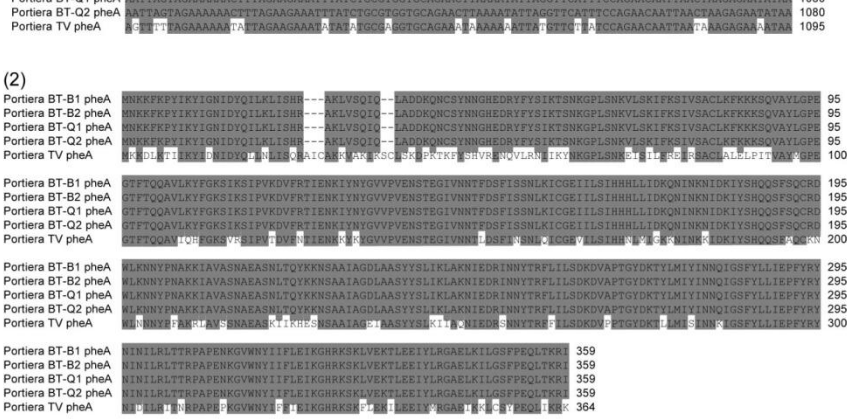

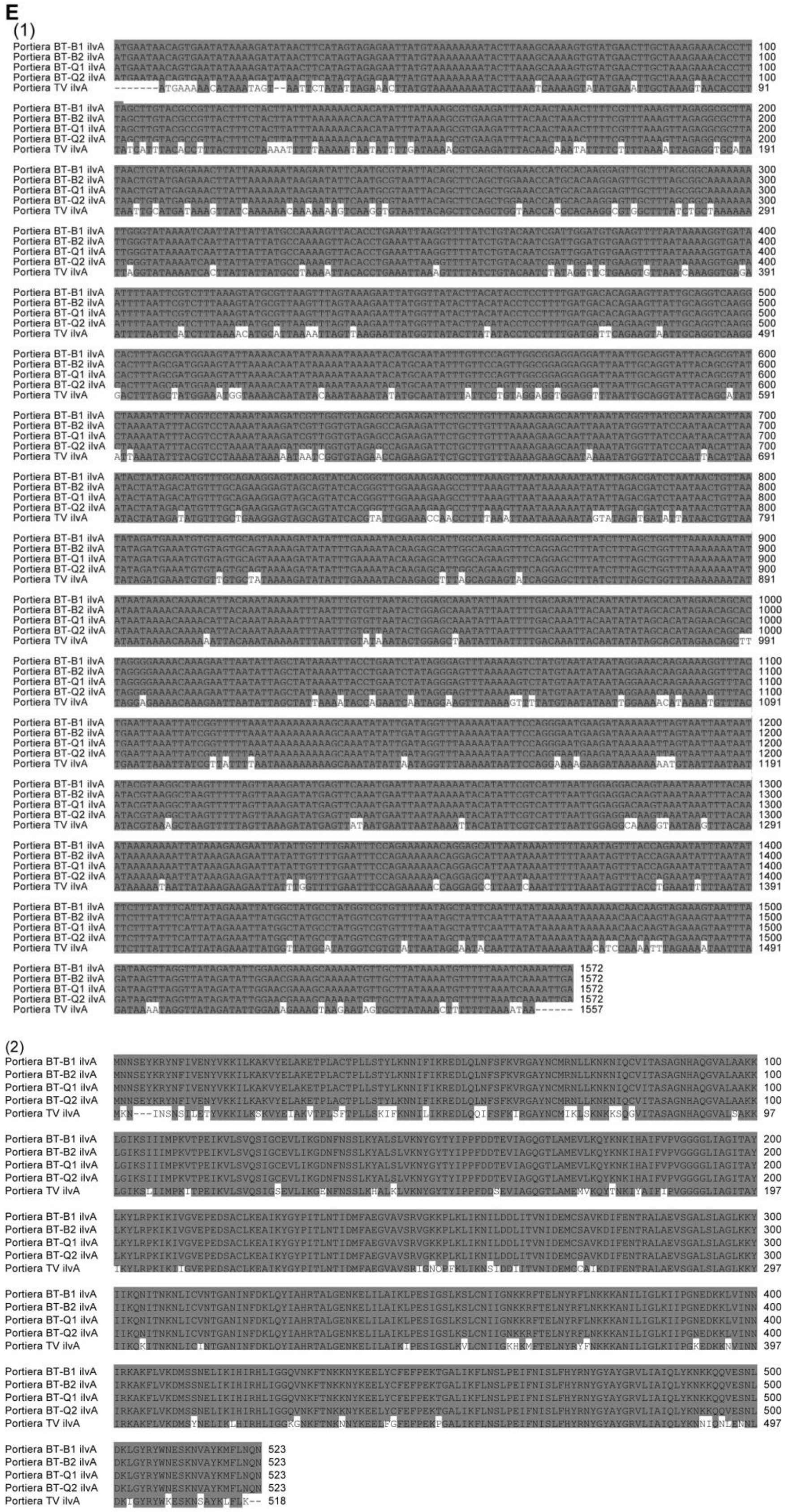
E
(1)
Portiera BT-B1 ilvA
Portiera BT-B2 ilvA
Portiera BT-Q1 ilvA
Portiera BT-Q2 ilvA
Portiera TV ilvA
(2)
