# Supplementary material for: Metabolic Coevolution in the Bacterial Symbiosis of Whiteflies and Related Plant Sap-Feeding Insects
Source: Genome Biol Evol. 2015 Sep 15;7(9):2635–47. doi: 10.1093/gbe/evv170 (PMC4607527; doi:10.1093/gbe/evv170)
Supplement: Supplementary Data [file supp_evv170_suppl_data.zip › Fig. S2-4.pdf]

Fig. S2

A

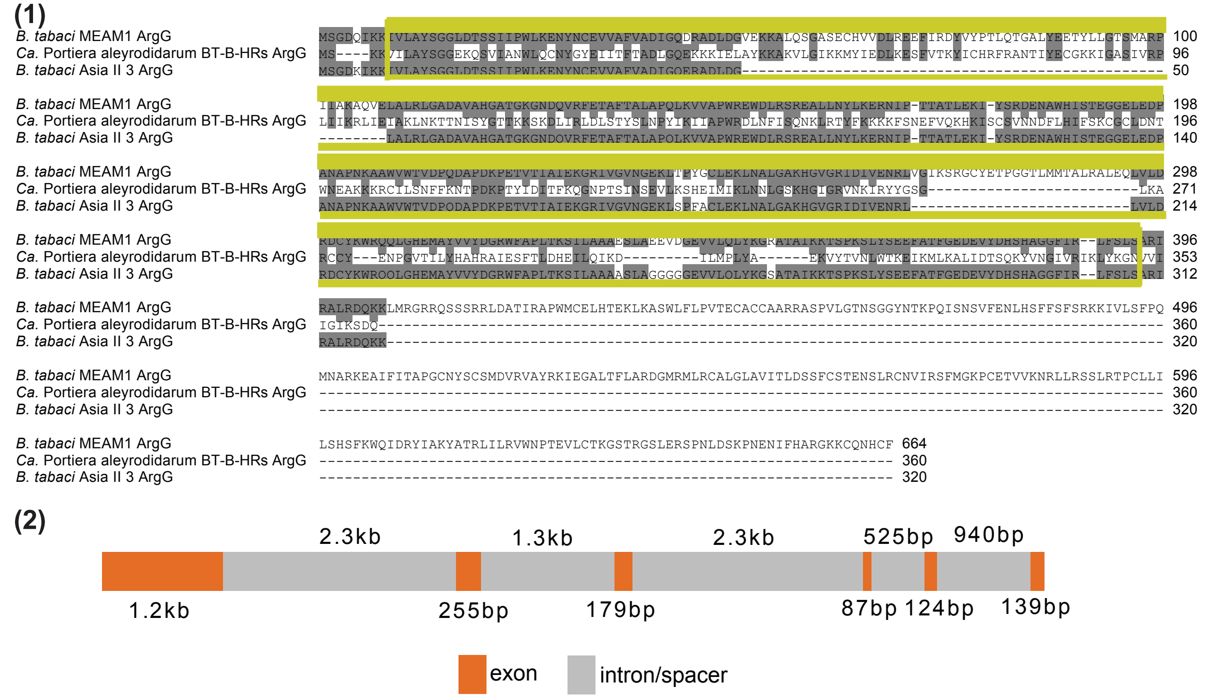
(1)
B. tabaci MEAM1 ArgG MSGDQIKKIVLAYSGGLDTSSIIPWLKENYNCEVVAFVADIGQDRADLDGVEKKALQSGASECHVVDLREEFIRDYVYPTLQTGALYEETYLLGTSMARP 100
Ca. Portiera aleyrodidarum BT-B-HRs ArgG MS----KKVILAYSGGEKQSVIANWLQCNYGYEIITFTADLGQEKKKIELAYKKAKVLGIKKMYIEDLKESFVTKYICHRFANTIYECGKKIGASIVRP 96
B. tabaci Asia II 3 ArgG MSGDKIKKIVLAYSGGLDTSSIIPWLKENYNCEVVAFVADIGQERADLDG-- 50
B. tabaci MEAM1 ArgG IIAKAQVELALRLGADAVAHGATGKGNDQVRFETAFTALAPQLKVVAPWREWDLRSREALLNYLKERNIP-TTATLEKI-YSRDENAWHISTEGGELEDP 198
Ca. Portiera aleyrodidarum BT-B-HRs ArgG LIIKRLIEIAKLNKTTNISYGTTKKSKDLIRLDLSTYSLNPYIKIIAPWRDLNFISQNKLRTYFKKKKFSNEFVQKHKISCSVNNDFLHIFSKCGCLDNT 196
B. tabaci Asia II 3 ArgG --------LALRLGADAVAHGATGKGNDQVRFETAFTALAPQLKVVAPWREWDLRSREALLNYLKERNIP-TTATLEKI-YSRDENAWHISTEGGELEDP 140
B. tabaci MEAM1 ArgG ANAPNKAAWVWTVDPQDAPDKPETVTIAIEKGRIVGVNGEKLTPYGCLEKLNALGAKHGVGRIDIVENRLVGIKSRGCYETPGGTLMMTALRALEQLVLD 298
Ca. Portiera aleyrodidarum BT-B-HRs ArgG WNEAKKKRCILSNFFKNTPDKPTYIDITFKQGNPTSINSEVLKSHEIMIKLNNLGSKHGIGRVNKIRYYGSG-------------------------LKA 271
B. tabaci Asia II 3 ArgG ANAPNKAAWVWTVDPQDAPDKPETVTIAIEKGRIVGVNGEKLSPFACLEKLNALGAKHGVGRIDIVENRL--------------------------LVLD 214
B. tabaci MEAM1 ArgG RDCYKWRQQLGHEMAYVVYDGRWFAPLTKSILAAAESLAEEVDGEVVLQLYKGRATATKKTSPKSLYSEEFATFGEDEVYDHSHAGGFIR--LFSLSARI 396
Ca. Portiera aleyrodidarum BT-B-HRs ArgG RCCY---ENPGVTILYHAHRAIESFTLDHEILQIKD---------ILMPLYA------EKVYTVNLWTKEIKMLKALIDTSQKYVNGIVRIKLYKGNVVI 353
B. tabaci Asia II 3 ArgG RDCYKWRQQLGHEMAYVVYDGRWFAPLTKSILAAAASLAGGGGGEVVLQLYKGSATATKKTSPKSLYSEEFATFGEDEVYDHSHAGGFIR--LFSLSARI 312
B. tabaci MEAM1 ArgG RALRDQKKLMRGRRQSSSRRLDATIRAPWMCELHTEKLKASWLFLPVTECACCAARRASPVLGTNSGGYNTKPQISNSVFENLHSFFSFSRKKIVLSFPQ 496
Ca. Portiera aleyrodidarum BT-B-HRs ArgG IGIKSDQ--- 360
B. tabaci Asia II 3 ArgG RALRDQKK-- 320
B. tabaci MEAM1 ArgG MNARKEAIFITAPGCNYSCSMDVRVAYRKIEGALTFLARDGMRMLRCALGLAVITLDSSFCSTENSLRCNVIRSFMGKPCETVVKNRLLRSSLRTPCLLI 596
Ca. Portiera aleyrodidarum BT-B-HRs ArgG -- 360
B. tabaci Asia II 3 ArgG -- 320
B. tabaci MEAM1 ArgG LSHSFKWQIDRYIAKYATRLILRVWNPTEVLCTKGSTRGSLERSPNLDSKPNENIFHARGKKCQNHCF 664
Ca. Portiera aleyrodidarum BT-B-HRs ArgG -- 360
B. tabaci Asia II 3 ArgG -- 320
(2)
2.3kb
1.3kb
2.3kb
525bp
940bp
1.2kb
255bp
179bp
87bp
124bp
139bp
exon
intron/spacer

Fig. S2

B

(1)

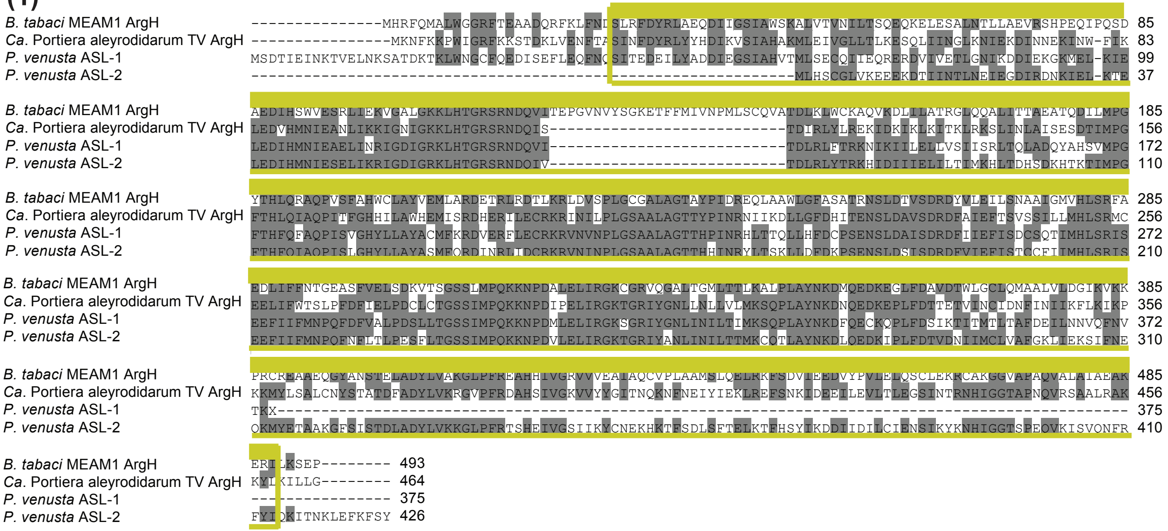
B. tabaci MEAM1 ArgH
Ca. Portiera aleyrodidarum TV ArgH
P. venusta ASL-1
P. venusta ASL-2
85
83
99
37
185
156
172
110
285
256
272
210
385
356
372
310
485
456
375
410
493
464
375
426

(2)

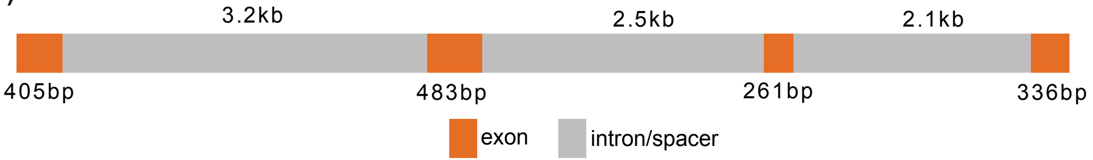
3.2kb
2.5kb
2.1kb
405bp
483bp
261bp
336bp
exon
intron/spacer

Fig. S2

C

(1)

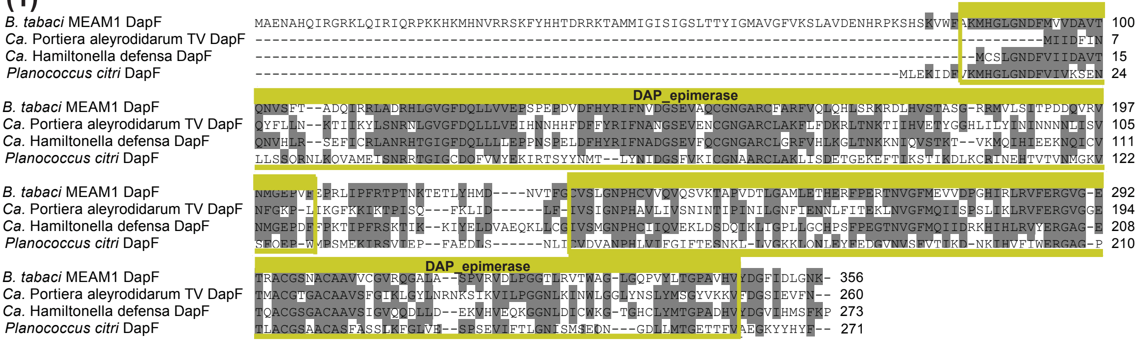

B. tabaci MEAM1 DapF
Ca. Portiera aleyrodidarum TV DapF
Ca. Hamiltonella defensa DapF
Planococcus citri DapF
MAENAHQIRGRKLQIRIQRPKKHKMHNVRRSKFYHHTDRRKTAMMIGISIGSLTTYIGMAVGFVKSLAVDENHRPKSHSKVWFAKMHGLGNDFMVVDAVT 100
---MIIDFIN 7
---MCSLGNDFVIIDAVT 15
--MLEKIDFVKMHGLGNDFVIVKSEN 24
DAP_epimerase
QNVSFT--ADQIRRLADRHLGVGFDQLLVVEPSPEPDVDFHYRIFNVDGSEVAQCGNGARCFARFVQLQHLSRKRDLHVSTASG-RRMVLSITPDDQVRV 197
QYFLLN--KTIIKYLSNRNLGVGFDQLLLVEIHNNHHFDFFYRIFNANGSEVENCGNGARCLAKFLFDKRLTNKTIIHVETYGGHLILYININNNNLISV 105
QNVHLR--SEFICRLANRHTGIGFDQLLLEPPNSPELDFHYRIFNADGSEVFQCGNGARCLGRFVHLKGLTNKKNIQVSTKT--VKMQIHIEEKNQICV 111
LLSSQRNLKQVAMEISNRRTGIGCDQFVVYEKIRTSYYNMT--LYNIDGSFVKICGNAARCLAKLISDETGEKEFTIKSTIKDLKCRINEHTVTVNMGKV 122
NMGEPVFEPRLIPFRTPTNKTETLYHMD----NVTFGCVSLGNPHCVVQVQSVKTAPVDTLGAMLETHERFPERTNVGFMEVVDPGHIRLRVFERGVG-E 292
NFGKP-LIKGFKKIKTPISQ---FKLID------LF-IVSIGNPHAVLIVSNINTIPINILGNFIENNLFITEKLNVGFMQIISPSLIKLRVFERGVGGE 194
NMGEPDFFPKTIPFRSKTIK--KIYELDVAEQKLLCGIVSMGNPHCIIQVEKLDSDQIKLIGPLLGCHPSFPEGTNVGFMQIIDRKHIHLRVYERGAG-E 208
SFQEP-WMPSMEKIRSVIEP--FAEDLS------NLICVDVANPHLVIFGIFTESNKL-LVGKKLQNLEYFEDGVNVSFVTIKD-NKIHVFIWERGAG-P 210
DAP_epimerase
TRACGSNACAAVVCGVRQGALA--SPVRVDLPGGTLRVTWAG-LGQPVYLTGPAVHVYDGFIDLGNK- 356
TMACGTGACAAVSFGIKLGYLNRNKSIKVILPGGNLKINWLGGLYNSLYMSGYVKKVFDGSIEVFN-- 260
TQACGSGACAAVSIGVQQDLLD--EKVHVEQKGGNLDICWKG-TGHCLYMTGPADHVYDGVIHMSFKP 273
TLACGSAACASFASSLKFGLVE--SPSEVIFTLGNISMSEQN---GDLLMTGETTFVAEGKYYHYF-- 271

(2)

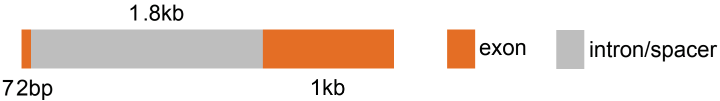

1.8kb
exon
intron/spacer
72bp
1kb

Fig. S2

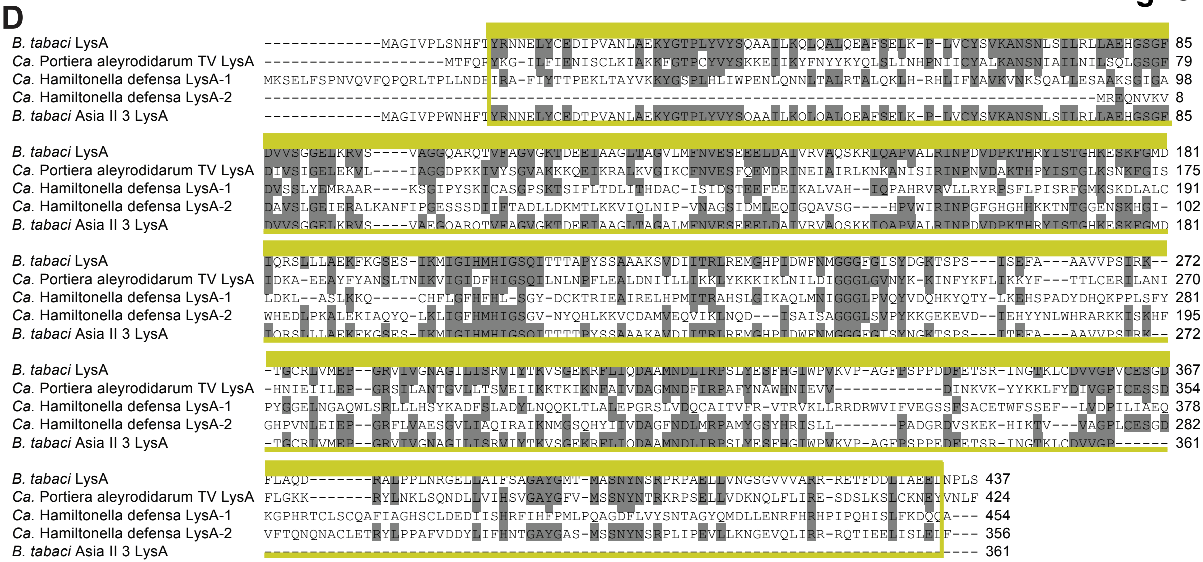
D
B. tabaci LysA
Ca. Portiera aleyrodidarum TV LysA
Ca. Hamiltonella defensa LysA-1
Ca. Hamiltonella defensa LysA-2
B. tabaci Asia II 3 LysA

Fig. S2

E

(1)

```
B. tabaci MEAM1 CM                          ---------------------------------------------------------------MDCACASWIRNNVKVNIVIFEEVENRDLILNQKELST 37
B. tabaci Asia II 3 CM                      ---------------------------------------------------------------------------------------------------- 1
Ca. Hamiltonella defensa CM                 ---------------------------------------------------------------------------------------------------- 1
Ca. Portiera aleyrodidarum BT-B-HRs CM      MNKKFKPYIKYIGNIDYQILKLISHRAKLVSQIQLADDKQNCSYNNGHEDRYFYSIKTSNKGPLSNKVLSKIFKSIVSACLKFKKKSQVAYLGPEGTFTQ 100

B. tabaci MEAM1 CM                          NSTWHKTGAKMGFIESALMVMAVSLCLFV------DVHPKSVA-----VCHPQTAEVCHPQTAEANAD---ASTGAMIILSLATTLNDRMEYMKDVAGDK 123
B. tabaci Asia II 3 CM                      ----------MGFIESALMAMA-SLCLFV------DVHSKSVA-----VCHPQTAEVCHPQT----AD---EC-G-------------RFD------GN- 50
Ca. Hamiltonella defensa CM                 ----------MSLIGINEIFLLINQRFY---------YMEDIA-----AYKAEK-QLAIEDL----EQ---EK---------------RIL------RN- 46
Ca. Portiera aleyrodidarum BT-B-HRs CM      QAVLKYFGKSIKSIPVKDVFRTIENKIYNYGVVPVENSTEGIVNNTFDSFISSNLKICGEIILSIHHHLLIDKQNINKNIDKIYSHQQSFSQCRDWLKNN 200

B. tabaci MEAM1 CM                          YKKGKPVEDLPREEKVMNSAVANASAEGLDPNSVRPFIQAQMDVAKDIQRTY--IAGWKVRPE-KWEPRALEEVR-EFISADDAK------ILRLSRQQL 213
B. tabaci Asia II 3 CM                      YD--HPVPG--RD--------AEQS-DGVHERRGRG--QAQMDVAKDIQRTY--IAGWKAR-----EVGA-EGSRGGQGAADDAK------ILRLSRQQL 121
Ca. Hamiltonella defensa CM                 -----AMLL------------AEQK--GITPNSIGSFFSALIHAAKLIQCRY--QSDWQKKSITKKHPQNLAQMRYKLIQLNDSL------IERVYQALI 119
Ca. Portiera aleyrodidarum BT-B-HRs CM      YPNAKKIAVASNAEASNLTQYKKNS-AAIAGDLAASY-YSLIKLAKNIEDRINNYTRFLILSDKDVAPTGYDKTY-LMIYINNQIGSFYLLIEPFYRYNI 297

B. tabaci MEAM1 CM                          ELGGFCEAYRAQ---IYRSLTAPHITDAHRKLLVD-TLFCVTLKQ----------------- 254
B. tabaci Asia II 3 CM                      TLGGFCEAYRAQ---IYRSLTAPYITDEHRKLLVD-TLFCVKLKQ----------------- 162
Ca. Hamiltonella defensa CM                 AGQKFEKTTSGS---MIKMIHHPQLSETHKRKIVT-ALQAIHLIQ----------------- 160
Ca. Portiera aleyrodidarum BT-B-HRs CM      NILRLTTRPAPENKGVWNYIIFLEIKGHRKSKLVEKTLEEIYLRGAELKILGSFPEQLTKRI 359
```

(2)

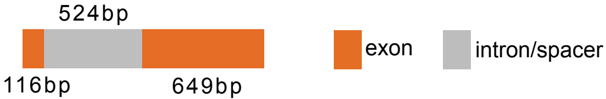

Fig. S2

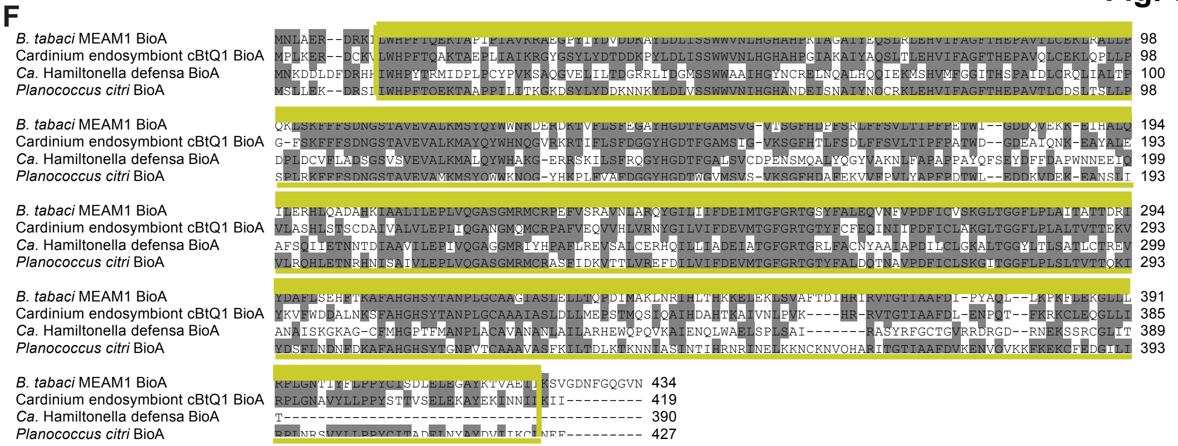
F
B. tabaci MEAM1 BioA
Cardinium endosymbiont cBtQ1 BioA
Ca. Hamiltonella defensa BioA
Planococcus citri BioA
98
98
100
98
194
193
199
193
294
293
299
293
391
385
389
393
434
419
390
427

Fig. S2

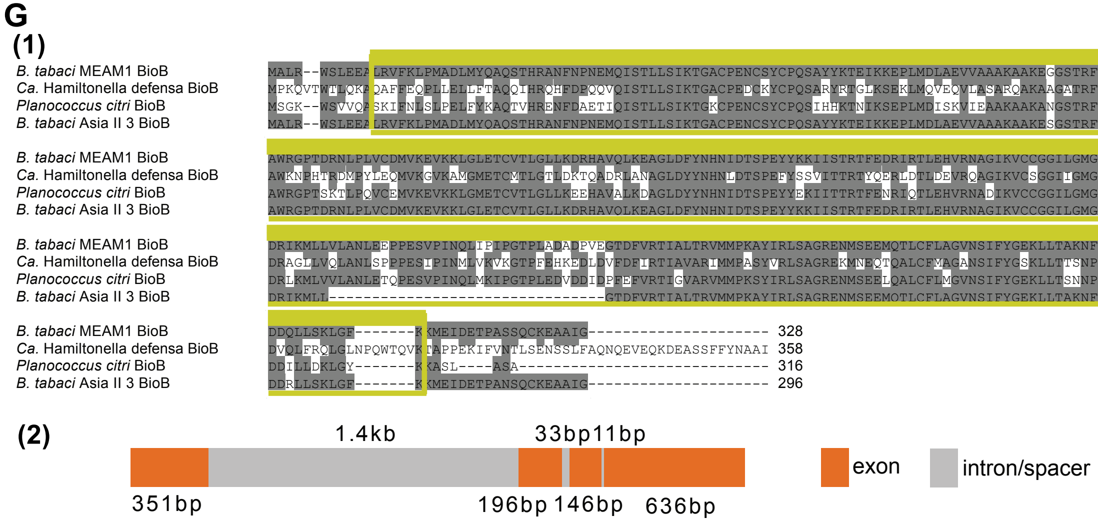
G
(1)
B. tabaci MEAM1 BioB
Ca. Hamiltonella defensa BioB
Planococcus citri BioB
B. tabaci Asia II 3 BioB
MALR--WSLEEALRVFKLPMADLMYQAQSTHRANFNPNEMQISTLLSIKTGACPENCSYCPQSAYYKTEIKKEPLMDLAEVVAAAKAAKEGGSTRFCMGA 98
MPKQVTWTLQKAQAFFEQPLLELLFTAQQIHRQHFDPQQVQISTLLSIKTGACPEDCKYCPQSARYRTGLKSEKLMQVEQVLASARQAKAAGATRFCMGA 100
MSGK--WSVVQASKIFNLSLPELFYKAQTVHRENFDAETIQISTLLSIKTGKCPENCSYCPQSIHHKTNIKSEPLMDISKVIEAAKAAKANGSTRFCMGA 98
MALR--WSLEEALRVFKLPMADLMYQAQSTHRANFNPNEMQISTLLSIKTGACPENCSYCPQSAYYKTEIKKEPLMDLAEVVAAAKAAKESGSTRFCMGA 98
AWRGPTDRNLPLVCDMVKEVKKLGLETCVTLGLLKDRHAVQLKEAGLDFYNHNIDTSPEYYKKIISTRTFEDRIRTLEHVRNAGIKVCCGGILGMGENTE 198
AWKNPHTRDMPYLEQMVKGVKAMGMETCMTLGTLDKTQADRLANAGLDYYNHNLDTSPEFYSSVITTRTYQERLDTLDEVRQAGIKVCSGGIIGMGESPK 200
AWRGPTSKTLPQVCEMVKEVKKLGMETCVTLGLLKEEHAVALKDAGLDYYNHNIDTSPEYYEKIITTRTFENRIQTLEHVRNADIKVCCGGILGMGETTE 198
AWRGPTDRNLPLVCDMVKEVKKLGLETCVTLGLLKDRHAVQLKEAGLDFYNHNIDTSPEYYKKIISTRTFEDRIRTLEHVRNAGIKVCCGGILGMGENTE 198
DRIKMLLVLANLEEPPESVPINQLIPIPGTPLADADPVEGTDFVRTIALTRVMMPKAYIRLSAGRENMSEEMQTLCFLAGVNSIFYGEKLLTAKNFQPSK 298
DRAGLLVQLANLSPPPESIPINMLVKVKGTPFEHKEDLDVFDFIRTIAVARIMMPASYVRLSAGREKMNEQTQALCFMAGANSIFYGSKLLTTSNPSEDK 300
DRLKMLVVLANLETQPESVPINQLMKIPGTPLEDVDDIDPFEFVRTIGVARVMMPKSYIRLSAGRENMSEELQALCFLMGVNSIFYGEKLLTSNNPAPIK 298
DRIKMLL---------------------------------GTDFVRTIALTRVMMPKAYIRLSAGRENMSEEMQTLCFLAGVNSIFYGEKLLTAKNFQPSK 266
DDQLLSKLGF-------KKMEIDETPASSQCKEAAIG--------------------- 328
DVQLFRQLGLNPQWTQVKTAPPEKIFVNTLSENSSLFAQNQEVEQKDEASSFFYNAAI 358
DDILLDKLGY-------KKASL----ASA----------------------------- 316
DDRLLSKLGF-------KKMEIDETPANSQCKEAAIG--------------------- 296
(2)
1.4kb
33bp
11bp
351bp
196bp
146bp
636bp
exon
intron/spacer

H

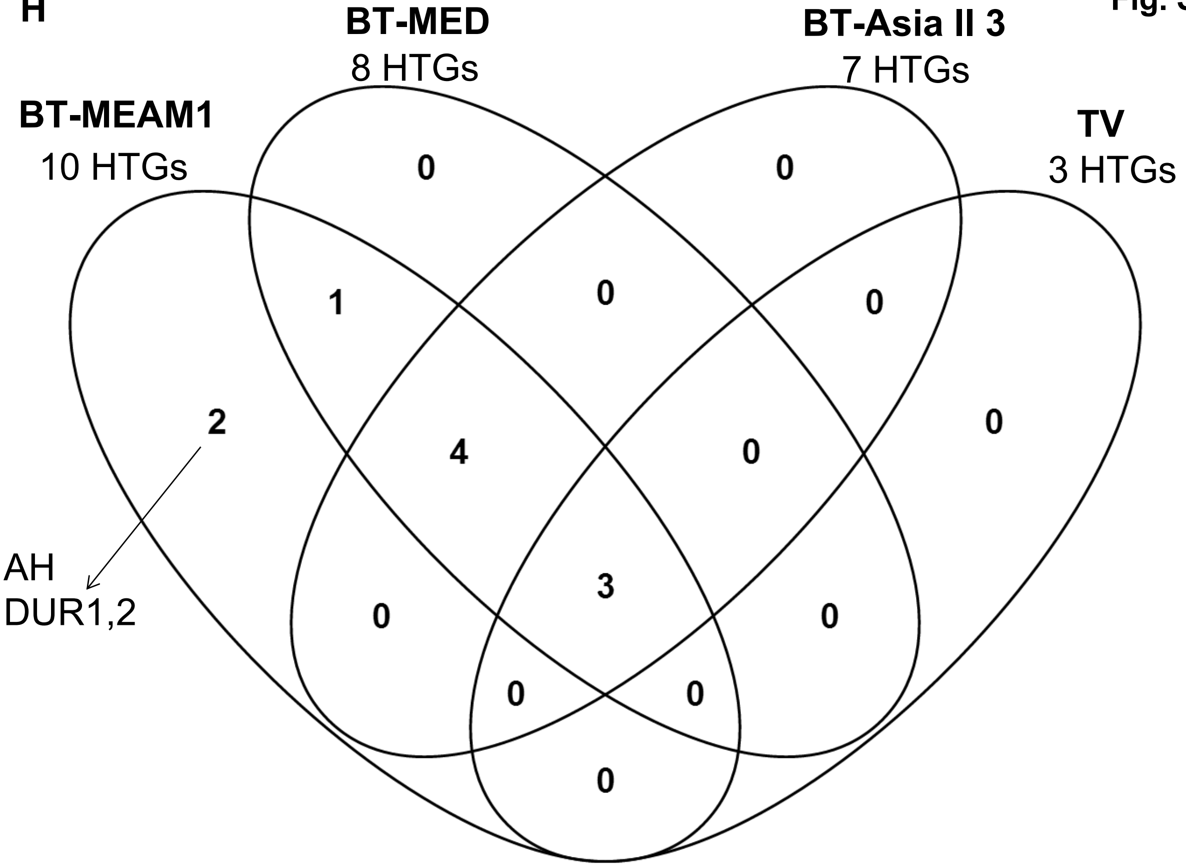
BT-MED
8 HTGs
BT-Asia II 3
7 HTGs
BT-MEAM1
10 HTGs
TV
3 HTGs
0
0
1
0
0
2
4
0
0
AH
DUR1,2
3
0
0
0
0
0

Fig. S3

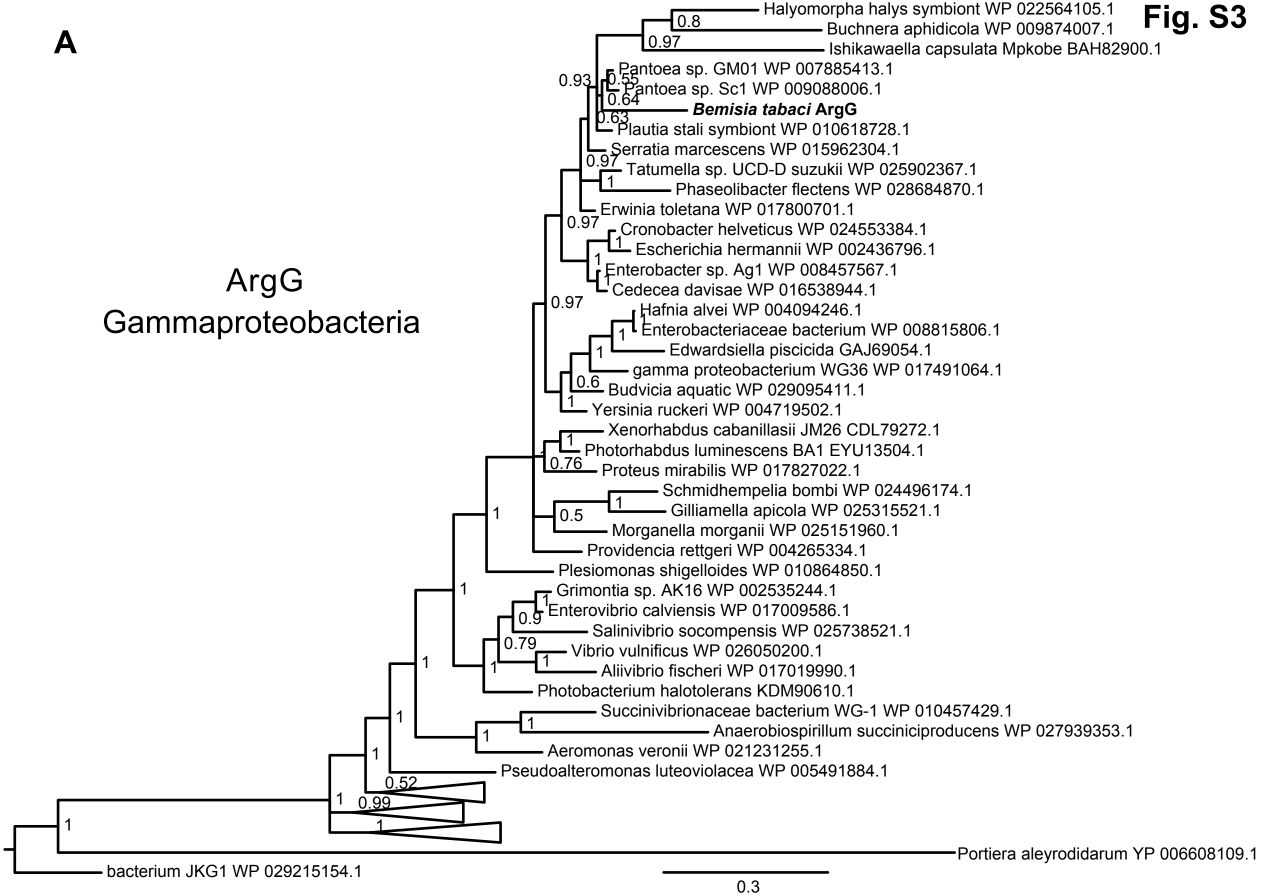
A
ArgG
Gammaproteobacteria
Halyomorpha halys symbiont WP 022564105.1
Buchnera aphidicola WP 009874007.1
Ishikawaella capsulata Mpkobe BAH82900.1
Pantoea sp. GM01 WP 007885413.1
Pantoea sp. Sc1 WP 009088006.1
Bemisia tabaci ArgG
Plautia stali symbiont WP 010618728.1
Serratia marcescens WP 015962304.1
Tatumella sp. UCD-D suzukii WP 025902367.1
Phaseolibacter flectens WP 028684870.1
Erwinia toletana WP 017800701.1
Cronobacter helveticus WP 024553384.1
Escherichia hermannii WP 002436796.1
Enterobacter sp. Ag1 WP 008457567.1
Cedecea davisae WP 016538944.1
Hafnia alvei WP 004094246.1
Enterobacteriaceae bacterium WP 008815806.1
Edwardsiella piscicida GAJ69054.1
gamma proteobacterium WG36 WP 017491064.1
Budvicia aquatic WP 029095411.1
Yersinia ruckeri WP 004719502.1
Xenorhabdus cabanillasii JM26 CDL79272.1
Photorhabdus luminescens BA1 EYU13504.1
Proteus mirabilis WP 017827022.1
Schmidhempelia bombi WP 024496174.1
Gilliamella apicola WP 025315521.1
Morganella morganii WP 025151960.1
Providencia rettgeri WP 004265334.1
Plesiomonas shigelloides WP 010864850.1
Grimontia sp. AK16 WP 002535244.1
Enterovibrio calviensis WP 017009586.1
Salinivibrio socompensis WP 025738521.1
Vibrio vulnificus WP 026050200.1
Aliivibrio fischeri WP 017019990.1
Photobacterium halotolerans KDM90610.1
Succinivibrionaceae bacterium WG-1 WP 010457429.1
Anaerobiospirillum succiniciproducens WP 027939353.1
Aeromonas veronii WP 021231255.1
Pseudoalteromonas luteoviolacea WP 005491884.1
Portiera aleyrodidarum YP 006608109.1
bacterium JKG1 WP 029215154.1
0.3
0.8
0.97
0.93
0.55
0.64
0.63
0.97
1
0.97
1
1
1
0.97
1
1
1
0.6
1
1
0.76
1
0.5
1
1
0.9
1
0.79
1
1
1
1
1
1
0.52
0.99
1
1
1

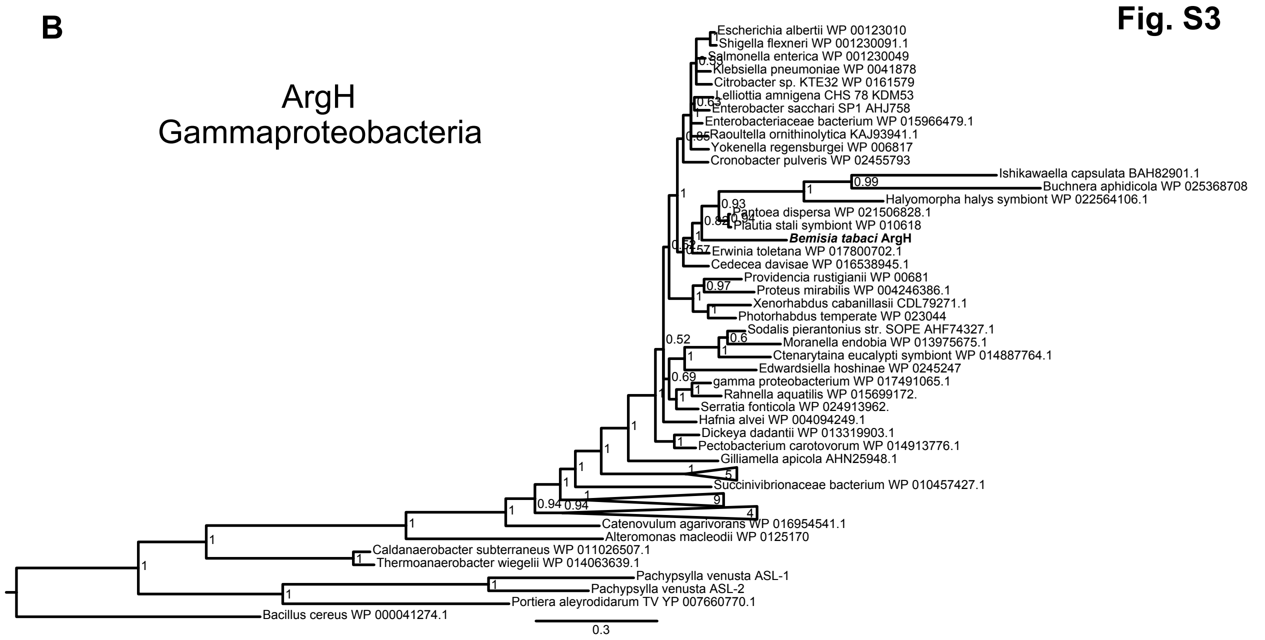
B
Fig. S3
ArgH
Gammaproteobacteria
Escherichia albertii WP 00123010
Shigella flexneri WP 001230091.1
Salmonella enterica WP 001230049
Klebsiella pneumoniae WP 0041878
Citrobacter sp. KTE32 WP 0161579
Lelliottia amnigena CHS 78 KDM53
Enterobacter sacchari SP1 AHJ758
Enterobacteriaceae bacterium WP 015966479.1
Raoultella ornithinolytica KAJ93941.1
Yokenella regensburgei WP 006817
Cronobacter pulveris WP 02455793
Ishikawaella capsulata BAH82901.1
Buchnera aphidicola WP 025368708
Halyomorpha halys symbiont WP 022564106.1
Pantoea dispersa WP 021506828.1
Plautia stali symbiont WP 010618
Bemisia tabaci ArgH
Erwinia toletana WP 017800702.1
Cedecea davisae WP 016538945.1
Providencia rustigianii WP 00681
Proteus mirabilis WP 004246386.1
Xenorhabdus cabanillasii CDL79271.1
Photorhabdus temperate WP 023044
Sodalis pierantonius str. SOPE AHF74327.1
Moranella endobia WP 013975675.1
Ctenarytaina eucalypti symbiont WP 014887764.1
Edwardsiella hoshinae WP 0245247
gamma proteobacterium WP 017491065.1
Rahnella aquatilis WP 015699172.
Serratia fonticola WP 024913962.
Hafnia alvei WP 004094249.1
Dickeya dadantii WP 013319903.1
Pectobacterium carotovorum WP 014913776.1
Gilliamella apicola AHN25948.1
Succinivibrionaceae bacterium WP 010457427.1
Catenovulum agarivorans WP 016954541.1
Alteromonas macleodii WP 0125170
Caldanaerobacter subterraneus WP 011026507.1
Thermoanaerobacter wiegelii WP 014063639.1
Pachypsylla venusta ASL-1
Pachypsylla venusta ASL-2
Portiera aleyrodidarum TV YP 007660770.1
Bacillus cereus WP 000041274.1
0.3

C

Fig. S3

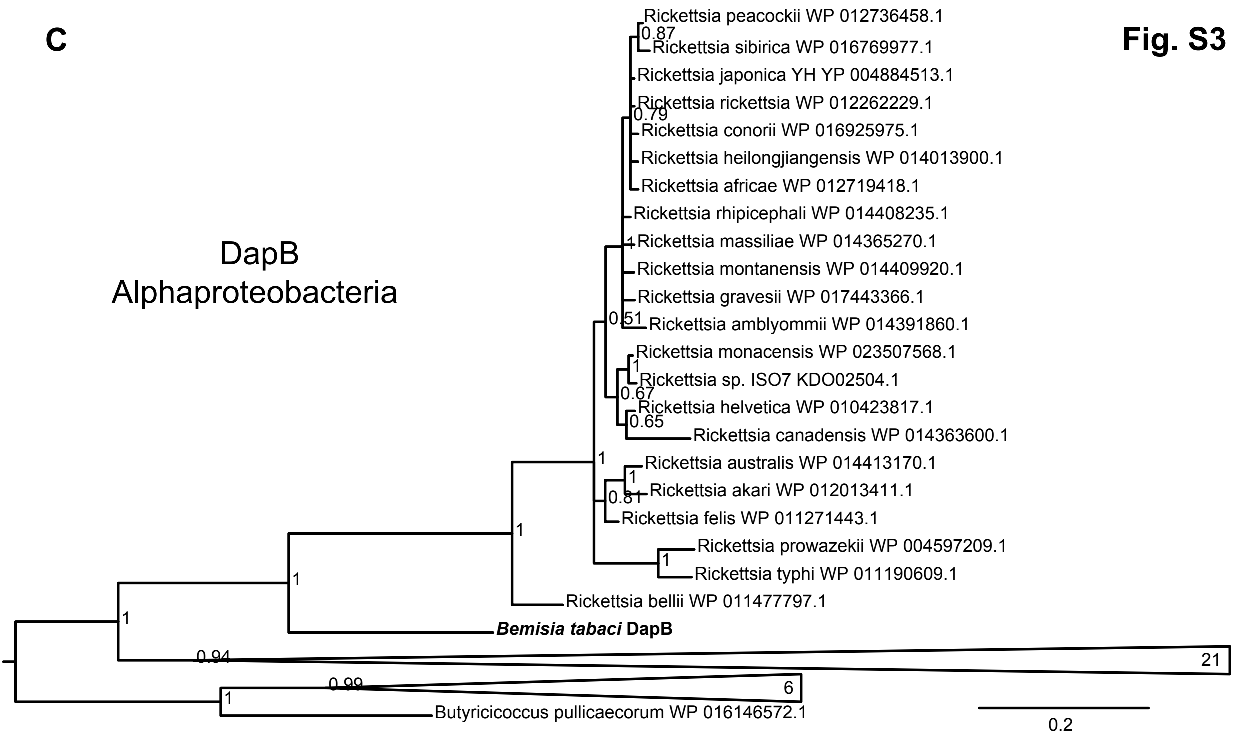
DapB
Alphaproteobacteria
Rickettsia peacockii WP 012736458.1
0.87
Rickettsia sibirica WP 016769977.1
Rickettsia japonica YH YP 004884513.1
Rickettsia rickettsia WP 012262229.1
0.79
Rickettsia conorii WP 016925975.1
Rickettsia heilongjiangensis WP 014013900.1
Rickettsia africae WP 012719418.1
Rickettsia rhipicephali WP 014408235.1
1
Rickettsia massiliae WP 014365270.1
Rickettsia montanensis WP 014409920.1
Rickettsia gravesii WP 017443366.1
0.51
Rickettsia amblyommii WP 014391860.1
Rickettsia monacensis WP 023507568.1
1
Rickettsia sp. ISO7 KDO02504.1
0.67
Rickettsia helvetica WP 010423817.1
0.65
Rickettsia canadensis WP 014363600.1
1
Rickettsia australis WP 014413170.1
1
Rickettsia akari WP 012013411.1
0.81
Rickettsia felis WP 011271443.1
1
Rickettsia prowazekii WP 004597209.1
1
Rickettsia typhi WP 011190609.1
1
Rickettsia bellii WP 011477797.1
1
Bemisia tabaci DapB
0.94
21
0.99
6
1
Butyricicoccus pullicaecorum WP 016146572.1
0.2

Fig. S3

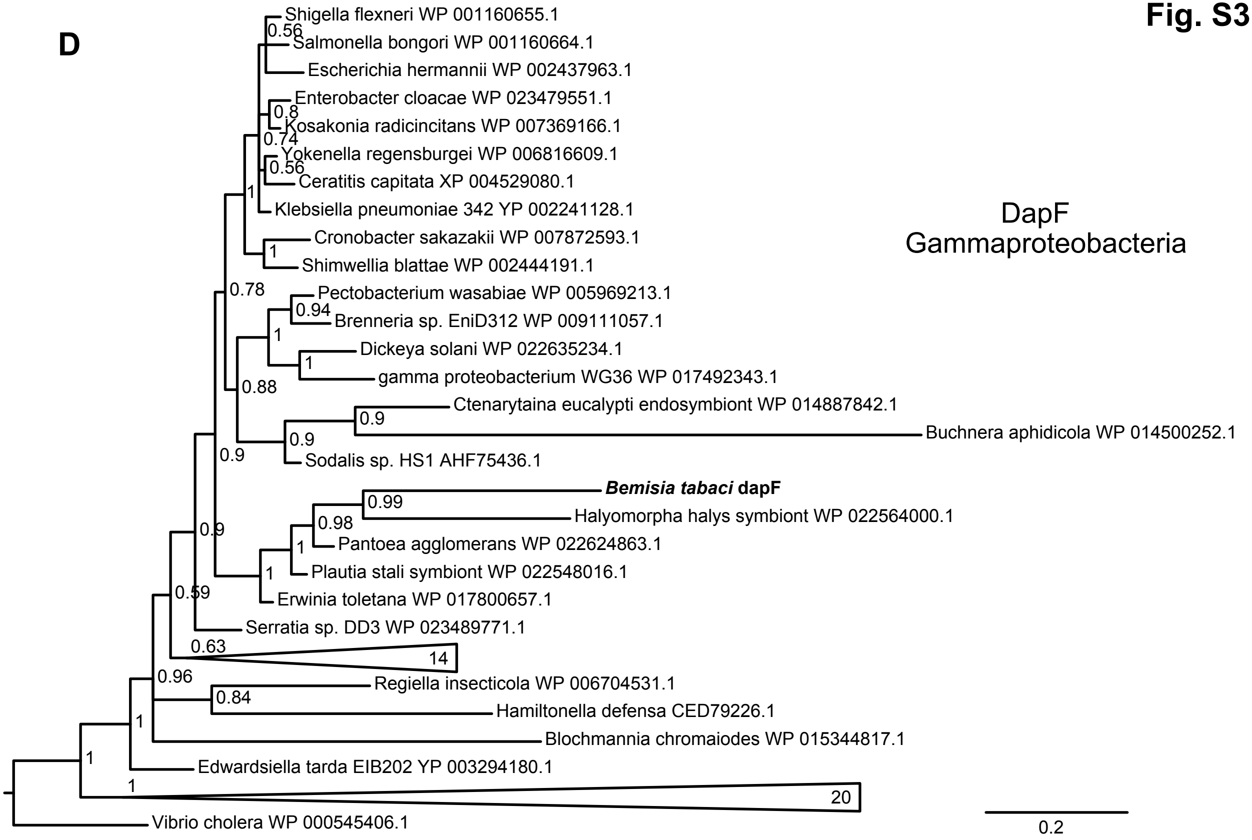
D
Shigella flexneri WP 001160655.1
0.56
Salmonella bongori WP 001160664.1
Escherichia hermannii WP 002437963.1
Enterobacter cloacae WP 023479551.1
0.8
Kosakonia radicincitans WP 007369166.1
0.74
Yokenella regensburgei WP 006816609.1
0.56
Ceratitis capitata XP 004529080.1
1
Klebsiella pneumoniae 342 YP 002241128.1
Cronobacter sakazakii WP 007872593.1
1
Shimwellia blattae WP 002444191.1
0.78
Pectobacterium wasabiae WP 005969213.1
0.94
Brenneria sp. EniD312 WP 009111057.1
1
Dickeya solani WP 022635234.1
1
gamma proteobacterium WG36 WP 017492343.1
0.88
Ctenarytaina eucalypti endosymbiont WP 014887842.1
0.9
Buchnera aphidicola WP 014500252.1
0.9
Sodalis sp. HS1 AHF75436.1
0.9
Bemisia tabaci dapF
0.99
Halyomorpha halys symbiont WP 022564000.1
0.98
Pantoea agglomerans WP 022624863.1
1
Plautia stali symbiont WP 022548016.1
1
0.9
Erwinia toletana WP 017800657.1
0.59
Serratia sp. DD3 WP 023489771.1
0.63
14
0.96
Regiella insecticola WP 006704531.1
0.84
Hamiltonella defensa CED79226.1
1
Blochmannia chromaiodes WP 015344817.1
1
Edwardsiella tarda EIB202 YP 003294180.1
1
20
Vibrio cholera WP 000545406.1
0.2
DapF
Gammaproteobacteria

Fig. S3

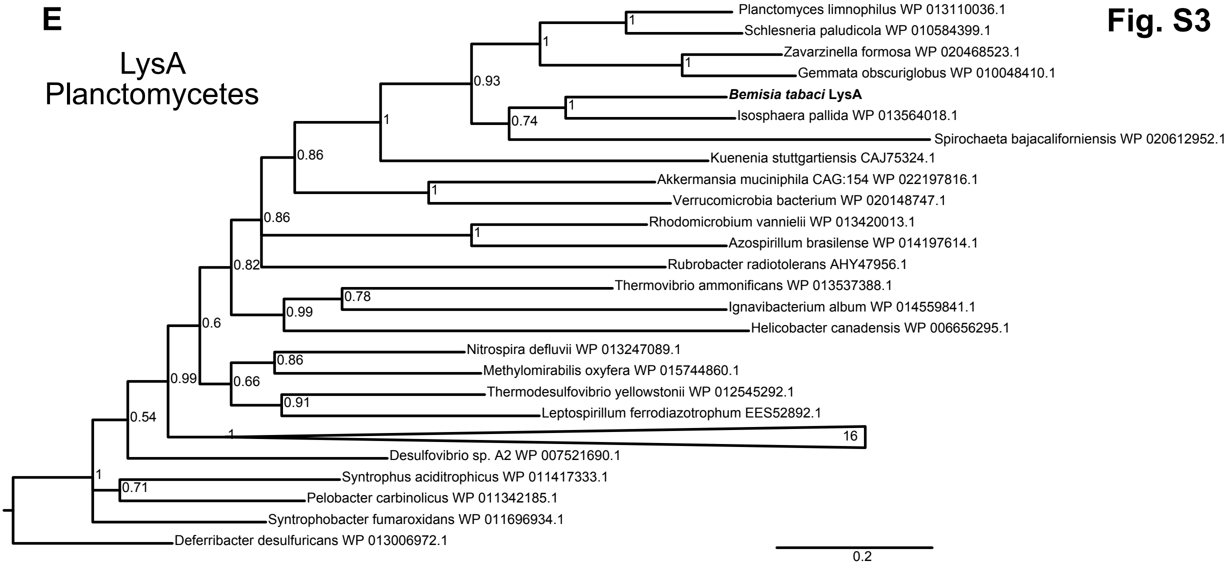
E
LysA
Planctomycetes
Planctomyces limnophilus WP 013110036.1
Schlesneria paludicola WP 010584399.1
Zavarzinella formosa WP 020468523.1
Gemmata obscuriglobus WP 010048410.1
Bemisia tabaci LysA
Isosphaera pallida WP 013564018.1
Spirochaeta bajacaliforniensis WP 020612952.1
Kuenenia stuttgartiensis CAJ75324.1
Akkermansia muciniphila CAG:154 WP 022197816.1
Verrucomicrobia bacterium WP 020148747.1
Rhodomicrobium vannielii WP 013420013.1
Azospirillum brasilense WP 014197614.1
Rubrobacter radiotolerans AHY47956.1
Thermovibrio ammonificans WP 013537388.1
Ignavibacterium album WP 014559841.1
Helicobacter canadensis WP 006656295.1
Nitrospira defluvii WP 013247089.1
Methylomirabilis oxyfera WP 015744860.1
Thermodesulfovibrio yellowstonii WP 012545292.1
Leptospirillum ferrodiazotrophum EES52892.1
16
Desulfovibrio sp. A2 WP 007521690.1
Syntrophus aciditrophicus WP 011417333.1
Pelobacter carbinolicus WP 011342185.1
Syntrophobacter fumaroxidans WP 011696934.1
Deferribacter desulfuricans WP 013006972.1
1
1
1
0.93
1
0.74
1
0.86
1
0.86
1
0.82
0.78
0.99
0.6
0.86
0.99
0.66
0.91
0.54
1
1
0.71
0.2

Fig. S3

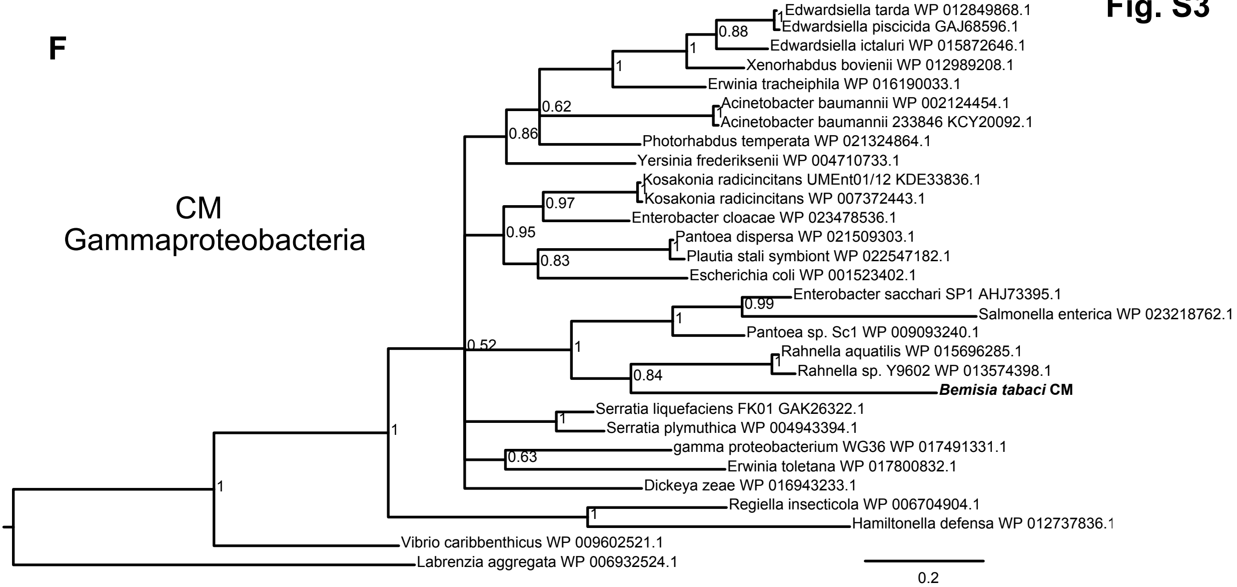
F
CM
Gammaproteobacteria
Edwardsiella tarda WP 012849868.1
Edwardsiella piscicida GAJ68596.1
Edwardsiella ictaluri WP 015872646.1
Xenorhabdus bovienii WP 012989208.1
Erwinia tracheiphila WP 016190033.1
Acinetobacter baumannii WP 002124454.1
Acinetobacter baumannii 233846 KCY20092.1
Photorhabdus temperata WP 021324864.1
Yersinia frederiksenii WP 004710733.1
Kosakonia radicincitans UMEnt01/12 KDE33836.1
Kosakonia radicincitans WP 007372443.1
Enterobacter cloacae WP 023478536.1
Pantoea dispersa WP 021509303.1
Plautia stali symbiont WP 022547182.1
Escherichia coli WP 001523402.1
Enterobacter sacchari SP1 AHJ73395.1
Salmonella enterica WP 023218762.1
Pantoea sp. Sc1 WP 009093240.1
Rahnella aquatilis WP 015696285.1
Rahnella sp. Y9602 WP 013574398.1
Bemisia tabaci CM
Serratia liquefaciens FK01 GAK26322.1
Serratia plymuthica WP 004943394.1
gamma proteobacterium WG36 WP 017491331.1
Erwinia toletana WP 017800832.1
Dickeya zeae WP 016943233.1
Regiella insecticola WP 006704904.1
Hamiltonella defensa WP 012737836.1
Vibrio caribbenthicus WP 009602521.1
Labrenzia aggregata WP 006932524.1
0.88
0.62
0.86
0.97
0.95
0.83
0.99
0.52
0.84
0.63
0.2

Fig. S3

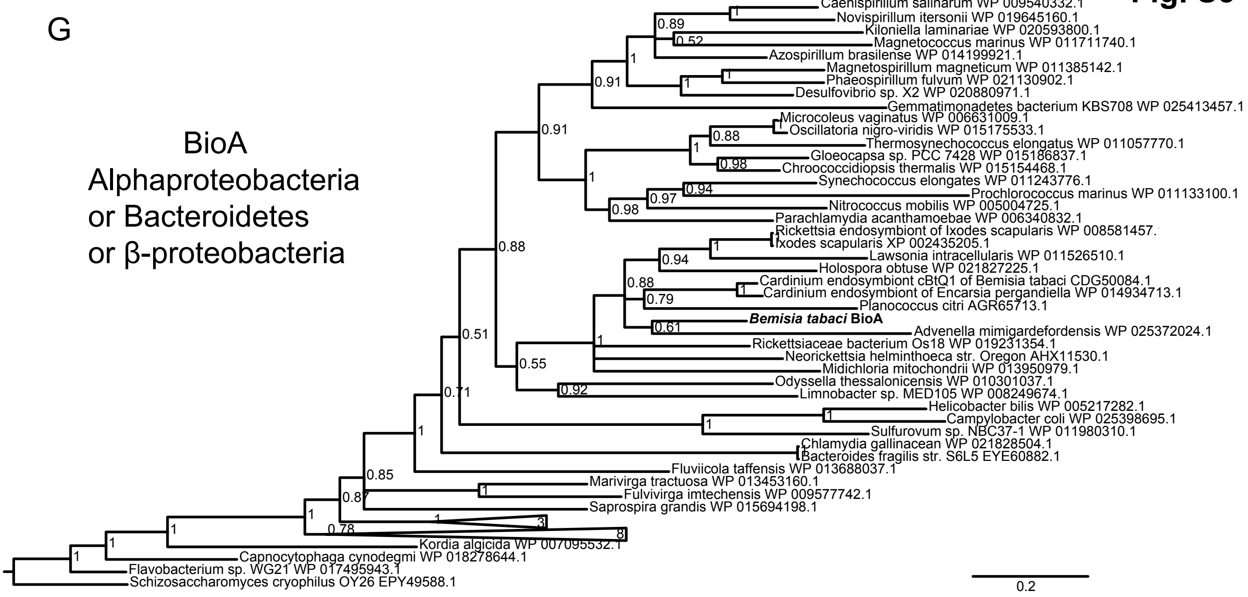
G
BioA
Alphaproteobacteria
or Bacteroidetes
or β-proteobacteria
Caenispirillum salinarum WP 009540332.1
Novispirillum itersonii WP 019645160.1
Kiloniella laminariae WP 020593800.1
Magnetococcus marinus WP 011711740.1
Azospirillum brasilense WP 014199921.1
Magnetospirillum magneticum WP 011385142.1
Phaeospirillum fulvum WP 021130902.1
Desulfovibrio sp. X2 WP 020880971.1
Gemmatimonadetes bacterium KBS708 WP 025413457.1
Microcoleus vaginatus WP 006631009.1
Oscillatoria nigro-viridis WP 015175533.1
Thermosynechococcus elongatus WP 011057770.1
Gloeocapsa sp. PCC 7428 WP 015186837.1
Chroococcidiopsis thermalis WP 015154468.1
Synechococcus elongates WP 011243776.1
Prochlorococcus marinus WP 011133100.1
Nitrococcus mobilis WP 005004725.1
Parachlamydia acanthamoebae WP 006340832.1
Rickettsia endosymbiont of Ixodes scapularis WP 008581457.
Ixodes scapularis XP 002435205.1
Lawsonia intracellularis WP 011526510.1
Holospora obtuse WP 021827225.1
Cardinium endosymbiont cBtQ1 of Bemisia tabaci CDG50084.1
Cardinium endosymbiont of Encarsia pergandiella WP 014934713.1
Planococcus citri AGR65713.1
Bemisia tabaci BioA
Advenella mimigardefordensis WP 025372024.1
Rickettsiaceae bacterium Os18 WP 019231354.1
Neorickettsia helminthoeca str. Oregon AHX11530.1
Midichloria mitochondrii WP 013950979.1
Odyssella thessalonicensis WP 010301037.1
Limnobacter sp. MED105 WP 008249674.1
Helicobacter bilis WP 005217282.1
Campylobacter coli WP 025398695.1
Sulfurovum sp. NBC37-1 WP 011980310.1
Chlamydia gallinacean WP 021828504.1
Bacteroides fragilis str. S6L5 EYE60882.1
Fluviicola taffensis WP 013688037.1
Marivirga tractuosa WP 013453160.1
Fulvivirga imtechensis WP 009577742.1
Saprospira grandis WP 015694198.1
Kordia algicida WP 007095532.1
Capnocytophaga cynodegmi WP 018278644.1
Flavobacterium sp. WG21 WP 017495943.1
Schizosaccharomyces cryophilus OY26 EPY49588.1
0.2

Fig. S3

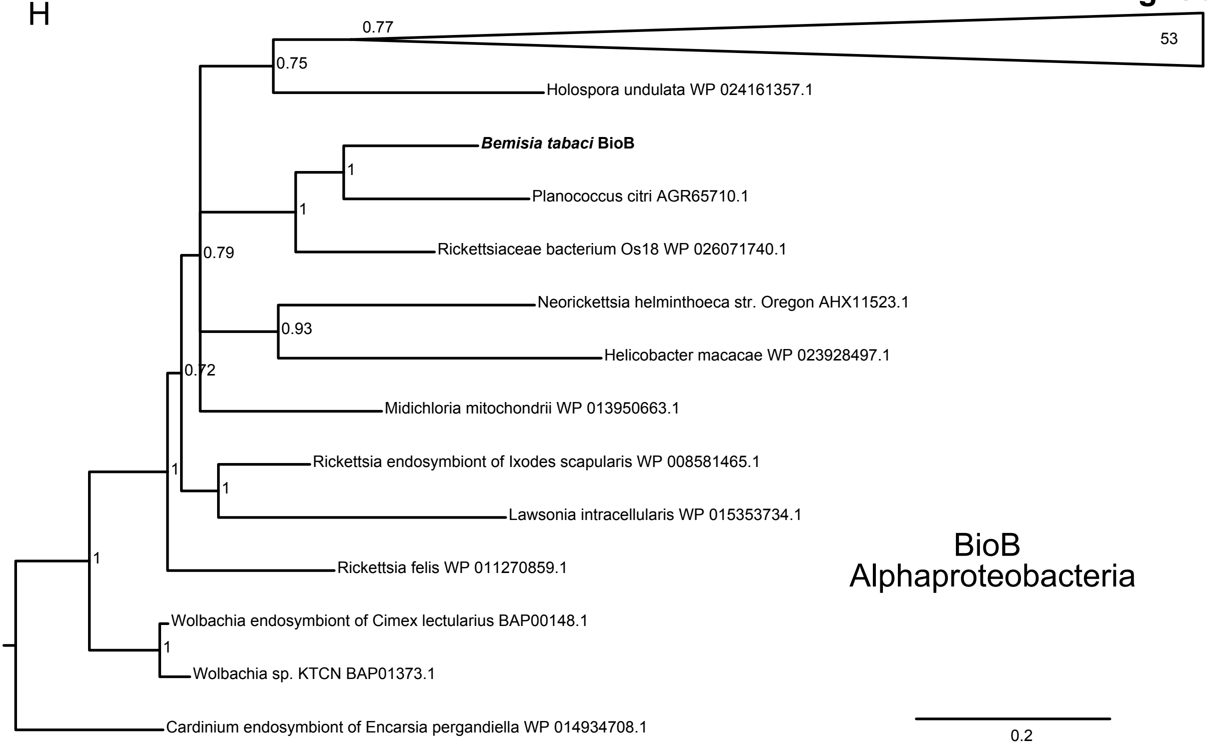
H
0.77
53
0.75
Holospora undulata WP 024161357.1
Bemisia tabaci BioB
1
Planococcus citri AGR65710.1
1
Rickettsiaceae bacterium Os18 WP 026071740.1
0.79
Neorickettsia helminthoeca str. Oregon AHX11523.1
0.93
Helicobacter macacae WP 023928497.1
0.72
Midichloria mitochondrii WP 013950663.1
Rickettsia endosymbiont of Ixodes scapularis WP 008581465.1
1
1
Lawsonia intracellularis WP 015353734.1
1
Rickettsia felis WP 011270859.1
BioB
Alphaproteobacteria
Wolbachia endosymbiont of Cimex lectularius BAP00148.1
1
Wolbachia sp. KTCN BAP01373.1
Cardinium endosymbiont of Encarsia pergandiella WP 014934708.1
0.2

Fig. S3

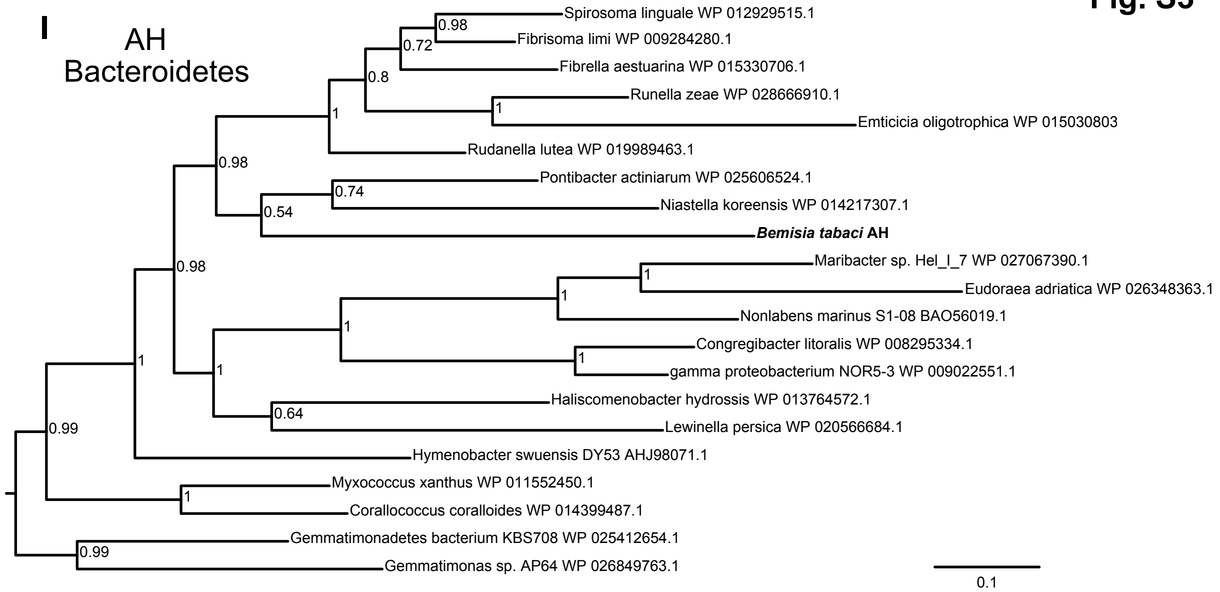
I
AH
Bacteroidetes
Spirosoma linguale WP 012929515.1
0.98
Fibrisoma limi WP 009284280.1
0.72
Fibrella aestuarina WP 015330706.1
0.8
Runella zeae WP 028666910.1
1
Emticicia oligotrophica WP 015030803
1
Rudanella lutea WP 019989463.1
0.98
Pontibacter actiniarum WP 025606524.1
0.74
Niastella koreensis WP 014217307.1
0.54
Bemisia tabaci AH
Maribacter sp. Hel_I_7 WP 027067390.1
0.98
1
Eudoraea adriatica WP 026348363.1
1
Nonlabens marinus S1-08 BAO56019.1
1
Congregibacter litoralis WP 008295334.1
1
1
gamma proteobacterium NOR5-3 WP 009022551.1
1
Haliscomenobacter hydrossis WP 013764572.1
0.64
Lewinella persica WP 020566684.1
0.99
Hymenobacter swuensis DY53 AHJ98071.1
Myxococcus xanthus WP 011552450.1
1
Corallococcus coralloides WP 014399487.1
Gemmatimonadetes bacterium KBS708 WP 025412654.1
0.99
Gemmatimonas sp. AP64 WP 026849763.1
0.1

Fig. S3

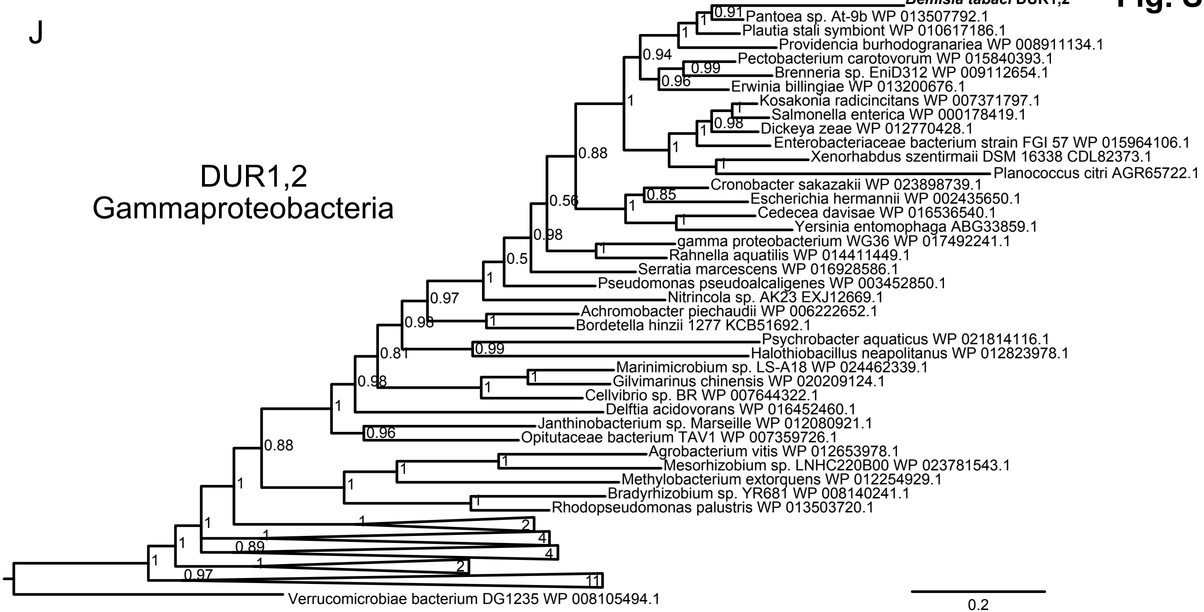

J
DUR1,2
Gammaproteobacteria
Bemisia tabaci DUR1,2
Pantoea sp. At-9b WP 013507792.1
Plautia stali symbiont WP 010617186.1
Providencia burhodogranariea WP 008911134.1
Pectobacterium carotovorum WP 015840393.1
Brenneria sp. EniD312 WP 009112654.1
Erwinia billingiae WP 013200676.1
Kosakonia radicincitans WP 007371797.1
Salmonella enterica WP 000178419.1
Dickeya zeae WP 012770428.1
Enterobacteriaceae bacterium strain FGI 57 WP 015964106.1
Xenorhabdus szentirmaii DSM 16338 CDL82373.1
Planococcus citri AGR65722.1
Cronobacter sakazakii WP 023898739.1
Escherichia hermannii WP 002435650.1
Cedecea davisae WP 016536540.1
Yersinia entomophaga ABG33859.1
gamma proteobacterium WG36 WP 017492241.1
Rahnella aquatilis WP 014411449.1
Serratia marcescens WP 016928586.1
Pseudomonas pseudoalcaligenes WP 003452850.1
Nitrincola sp. AK23 EXJ12669.1
Achromobacter piechaudii WP 006222652.1
Bordetella hinzii 1277 KCB51692.1
Psychrobacter aquaticus WP 021814116.1
Halothiobacillus neapolitanus WP 012823978.1
Marinimicrobium sp. LS-A18 WP 024462339.1
Gilvimarinus chinensis WP 020209124.1
Cellvibrio sp. BR WP 007644322.1
Delftia acidovorans WP 016452460.1
Janthinobacterium sp. Marseille WP 012080921.1
Opitutaceae bacterium TAV1 WP 007359726.1
Agrobacterium vitis WP 012653978.1
Mesorhizobium sp. LNHC220B00 WP 023781543.1
Methylobacterium extorquens WP 012254929.1
Bradyrhizobium sp. YR681 WP 008140241.1
Rhodopseudomonas palustris WP 013503720.1
Verrucomicrobiae bacterium DG1235 WP 008105494.1
0.2

Fig. S4

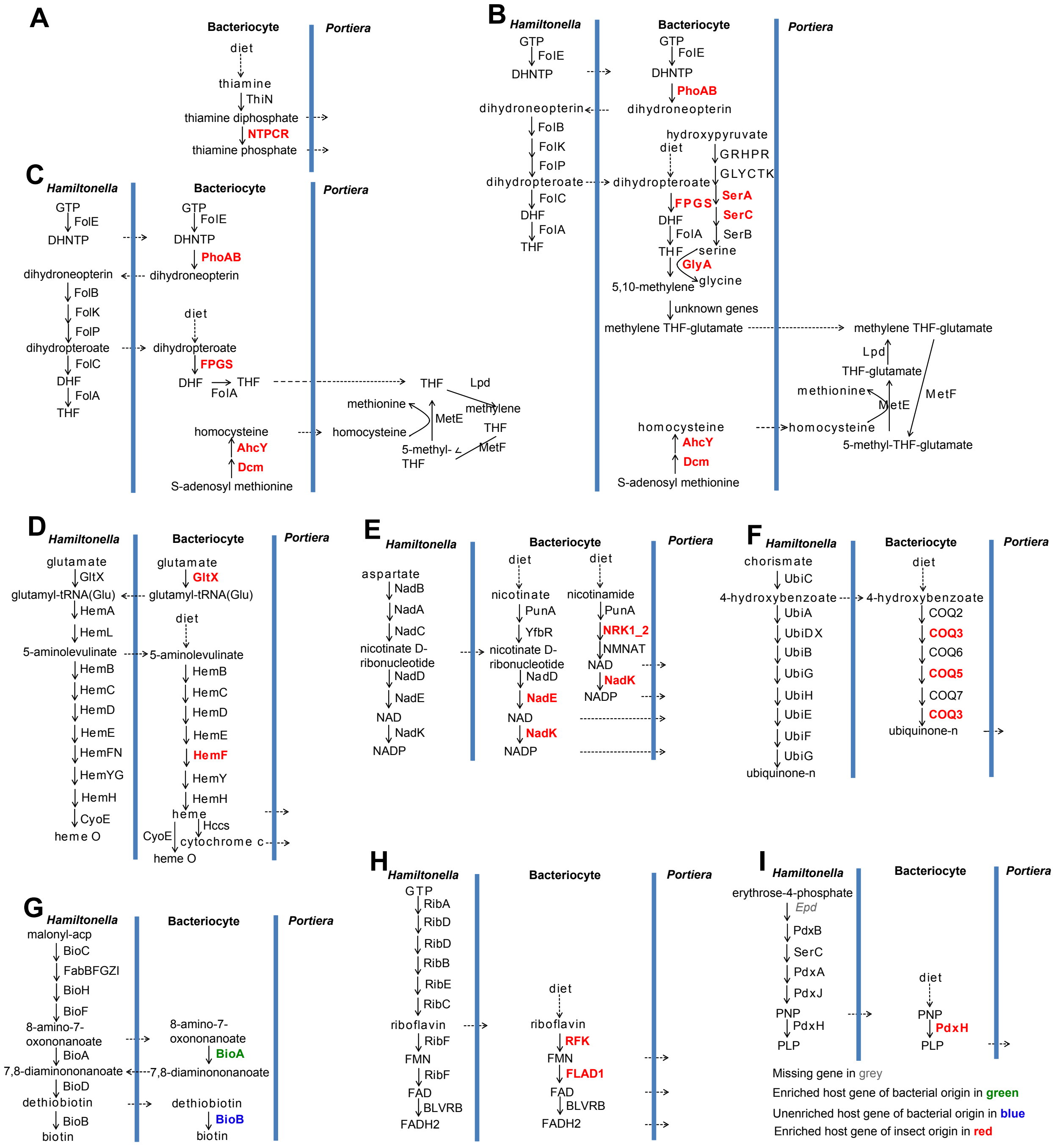
A
Bacteriocyte
Portiera
diet
thiamine
ThiN
thiamine diphosphate
NTPCR
thiamine phosphate
B
Hamiltonella
Bacteriocyte
Portiera
GTP
FolE
DHNTP
PhoAB
dihydroneopterin
FolB
FolK
FolP
dihydropteroate
FolC
DHF
FolA
THF
hydroxypyruvate
diet
GRHPR
GLYCTK
SerA
FPGS
SerC
SerB
serine
GlyA
glycine
5,10-methylene
unknown genes
methylene THF-glutamate
Lpd
THF-glutamate
methionine
MetE
MetF
homocysteine
AhcY
Dcm
S-adenosyl methionine
5-methyl-THF-glutamate
C
Hamiltonella
Bacteriocyte
Portiera
GTP
FolE
DHNTP
PhoAB
dihydroneopterin
FolB
FolK
FolP
diet
dihydropteroate
FPGS
FolC
DHF
FolA
THF
Lpd
methionine
MetE
methylene
THF
MetF
5-methyl-THF
homocysteine
AhcY
Dcm
S-adenosyl methionine
D
Hamiltonella
Bacteriocyte
Portiera
glutamate
GltX
glutamyl-tRNA(Glu)
HemA
HemL
diet
5-aminolevulinate
HemB
HemC
HemD
HemE
HemFN
HemF
HemYG
HemY
HemH
CyoE
heme O
heme
Hccs
cytochrome c
E
Hamiltonella
Bacteriocyte
Portiera
aspartate
NadB
NadA
NadC
nicotinate D-ribonucleotide
NadD
NadE
NAD
NadK
NADP
diet
nicotinate
PunA
YfbR
nicotinamide
NRK1_2
NMNAT
F
Hamiltonella
Bacteriocyte
Portiera
chorismate
UbiC
4-hydroxybenzoate
UbiA
UbiDX
UbiB
UbiG
UbiH
UbiE
UbiF
UbiG
ubiquinone-n
diet
COQ2
COQ3
COQ6
COQ5
COQ7
COQ3
G
Hamiltonella
Bacteriocyte
Portiera
malonyl-acp
BioC
FabBFGZI
BioH
BioF
8-amino-7-oxononanoate
BioA
7,8-diaminononanoate
BioD
dethiobiotin
BioB
biotin
H
Hamiltonella
Bacteriocyte
Portiera
GTP
RibA
RibD
RibD
RibB
RibE
RibC
riboflavin
RibF
FMN
FAD
BLVRB
FADH2
diet
RFK
FLAD1
I
Hamiltonella
Bacteriocyte
Portiera
erythrose-4-phosphate
Epd
PdxB
SerC
PdxA
PdxJ
PNP
PdxH
PLP
diet
Missing gene in grey
Enriched host gene of bacterial origin in green
Unenriched host gene of bacterial origin in blue
Enriched host gene of insect origin in red

Fig. S4

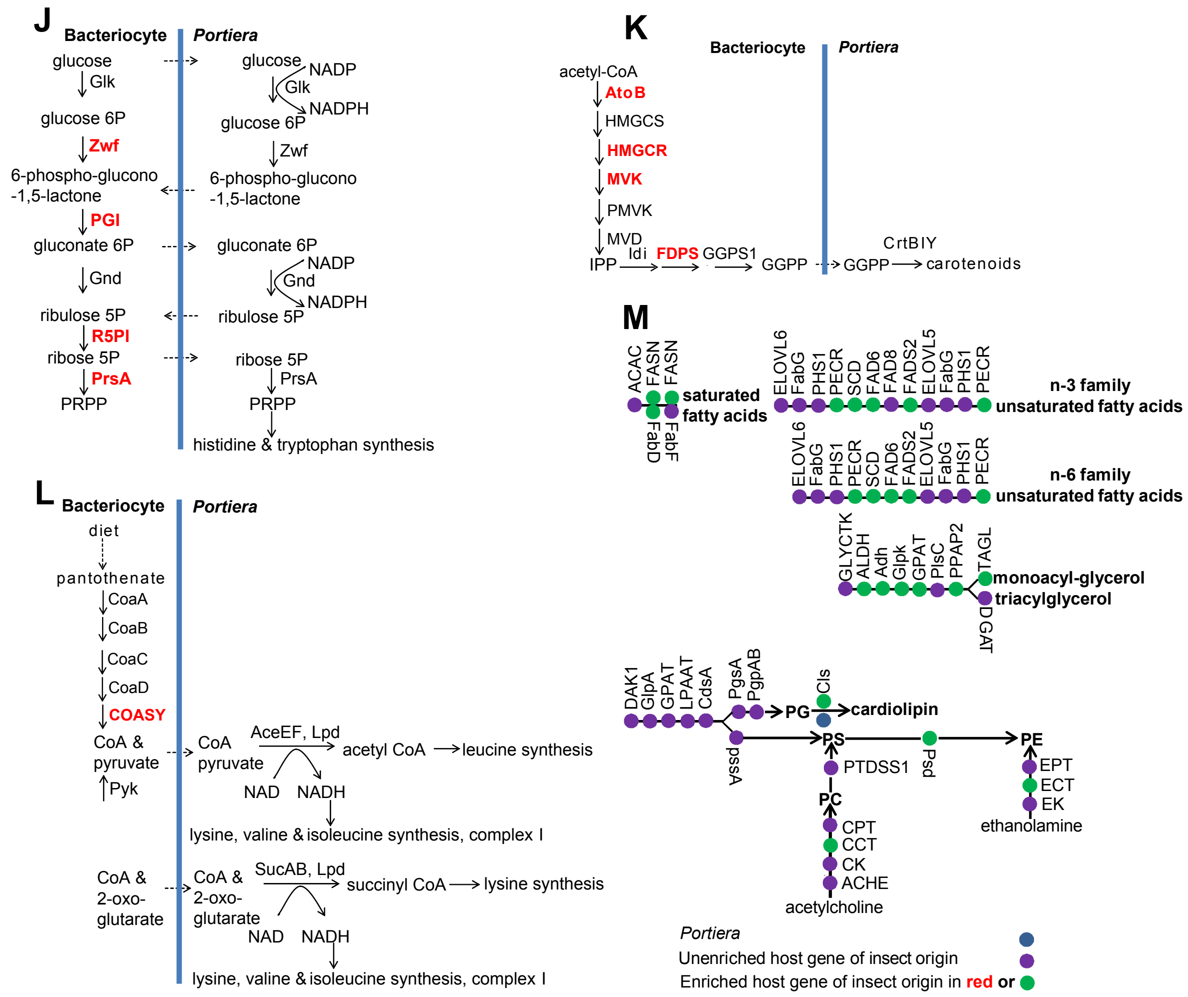

J
Bacteriocyte
Portiera
glucose
Glk
glucose 6P
Zwf
6-phospho-glucono
-1,5-lactone
PGI
gluconate 6P
Gnd
ribulose 5P
R5PI
ribose 5P
PrsA
PRPP
glucose
NADP
Glk
NADPH
glucose 6P
Zwf
6-phospho-glucono
-1,5-lactone
gluconate 6P
NADP
Gnd
NADPH
ribulose 5P
ribose 5P
PrsA
PRPP
histidine & tryptophan synthesis
K
Bacteriocyte
Portiera
acetyl-CoA
AtoB
HMGCS
HMGCR
MVK
PMVK
MVD
IPP
Idi
FDPS
GGPS1
GGPP
GGPP
CrtBIY
carotenoids
L
Bacteriocyte
Portiera
diet
pantothenate
CoaA
CoaB
CoaC
CoaD
COASY
CoA &
pyruvate
Pyk
CoA
pyruvate
AceEF, Lpd
acetyl CoA
leucine synthesis
NAD
NADH
lysine, valine & isoleucine synthesis, complex I
CoA &
2-oxo-
glutarate
CoA &
2-oxo-
glutarate
SucAB, Lpd
succinyl CoA
lysine synthesis
NAD
NADH
lysine, valine & isoleucine synthesis, complex I
M
ACAC
FASN
FASN
FabD
FabF
saturated
fatty acids
ELOVL6
FabG
PHS1
PECR
SCD
FAD6
FAD8
FADS2
ELOVL5
FabG
PHS1
PECR
n-3 family
unsaturated fatty acids
ELOVL6
FabG
PHS1
PECR
SCD
FAD6
FADS2
ELOVL5
FabG
PHS1
PECR
n-6 family
unsaturated fatty acids
GLYCTK
ALDH
Adh
Glpk
GPAT
PlsC
PPAP2
TAGL
DGAT
monoacyl-glycerol
triacylglycerol
DAK1
GlpA
GPAT
LPAAT
CdsA
PgsA
PgpAB
PG
Cls
cardiolipin
PssA
PS
Psd
PE
PTDSS1
PC
CPT
CCT
CK
ACHE
acetylcholine
EPT
ECT
EK
ethanolamine
Portiera
Unenriched host gene of insect origin
Enriched host gene of insect origin in red or
