# Supplementary material for: Metabolic Coevolution in the Bacterial Symbiosis of Whiteflies and Related Plant Sap-Feeding Insects
Source: Genome Biol Evol. 2015 Sep 15;7(9):2635–47. doi: 10.1093/gbe/evv170 (PMC4607527; doi:10.1093/gbe/evv170)
Supplement: Supplementary Data [file supp_evv170_suppl_data.zip › Table S1-S4.docx]

**Table S1 qRT-PCR primers**

| *Gene name* | *Description* | *Forward (5'-3')* | *Reverse (5'-3')* | *Amplicon length (bp)* | *Ampliﬁcation efﬁciencies (%)* |
| --- | --- | --- | --- | --- | --- |
| 1. qRT-PCR for whitefly genes | | | | | |
| *GOGAT* | glutamate synthase | GCTCTCTTGACATTCACCGA | TGCAGGGTCCGTATCATAAA | 141 | 100.52 |
| *aspC* | aspartate aminotransferase | CAGCACTTGTGGTTTCGATT | GGATCAACGCCTGTAGGATT | 111 | 95.3 |
| *ilvE* | branched-chain-amino-acid aminotransferase | GCAGGATGAGACCATTCAAA | AAATCATGCTGTCACCGAAG | 107 | 95.43 |
| *serA* | D-3-phosphoglycerate dehydrogenase | GGGTGTGTTGAGAACCAAGA | TGCCTCTCGGTTGAGTGTAG | 100 | 100.64 |
| *atoB* | acetyl-CoA C-acetyltransferase | CAGCCTGCTTTCTGCAATAC | TCTTATGGACCGAGCAACTG | 144 | 101.93 |
| *FDPS* | farnesyl diphosphate synthase | CTTGCAAAGAGCAACACCAT | AGATTTGAGGCAGACCCAAC | 119 | 105.02 |
| *MVK* | mevalonate kinase | TAAGCTGTTGGGCACTTCAT | TACACTGGAAACCACCCTCA | 138 | 107.7 |
| *SDR16C5* | all-trans-retinol dehydrogenase | GCACCAAGTTACGTAGCGAA | GAGCTGACAAGGAATGCTGA | 78 | 98.82 |
| *fabD* | [acyl-carrier-protein] S-malonyltransferase | ACCTGTTTGGCATCGTTGTA | CACACGGATCTGATGAAACC | 128 | 101.63 |
| *PECR* | trans-2-enoyl-CoA reductase | TGCCGATCATATCCTCATTG | AAGATGTCGCATCACCTCTG | 130 | 100.6 |
| *FAD6* | delta-12 desaturase | TTTCGACTATGCCGCACTAC | CTCCACAAACACGCACTCTT | 137 | 102.38 |
| *psd* | phosphatidylserine decarboxylase | TGTAGCACCAACAGCAGTCA | AGAGCTGGTCCCTGAACTGT | 97 | 105.52 |
| *GPAT* | glycerol-3-phosphate O-acyltransferase 3/4 | GTCTGGTACTTACCGCCCAT | GTCGACTAAGCCACCCTGTT | 102 | 99.42 |
| *HADHA* | enoyl-CoA hydratase | CTATTTCAGGCTGGCCAAAT | CACTGGAACAAGGTTAGCGA | 142 | 106.33 |
| *HMG-CoA reductase* | hydroxymethylglutaryl-CoA reductase (NADPH) | GCGAGCGGCACATATACTAA | GTTCTGGGAGCTTGTTGTGA | 104 | 99.4 |
| *lysA* | diaminopimelate decarboxylase | CTGTTGGTCAATGGATCAGG | AGCTCCTCGGCAATTAAGTC | 74 | 107.9 |
| *argH* | argininosuccinate lyase | AAGATGTTGACGGTGACGAG | ACTCCTTGCGCTTCGACTAC | 88 | 104.76 |
| *dapF* | diaminopimelate epimerase | GCATGGTCCTCTCCATCAC | ACCGAATGTCACGTTATCCA | 140 | 102.2 |
| *CM* | chorismate mutase | TTCCTATGCGCGTCAGTAAT | CAGCTTGAACTTGGAGGGTT | 89 | 105.4 |
| *PDHA1* | pyruvate dehydrogenase | GGAGGGAGGAGTATGCTTCA | TTCATCAATTTGGCGATGTT | 90 | 100.47 |
| *PDHA1* | pyruvate dehydrogenase | CAAAGTATCCCGAGGCTTGT | GAAGGTCATGAACTCGCAAA | 139 | 105.6 |
| *argG* | argininosuccinate synthase | ACCTTACGGGTGTTTGGAGA | GACTTAATGCCCACCAACCT | 96 | 106.2 |
| *AH* | amidohydrolase | ATCCAGAGCCGGAAAGAGTA | TGCAAATGGTCACTCCACTT | 75 | 93.52 |
| *bioA* | adenosylmethionine-8-amino-7-oxononanoate aminotransferase | ATGAGGATGTGTCGTCCAGA | CAAAGTTCACCTGCTCCAAA | 139 | 103.58 |
| *bioB* | biotin synthase | TCAACCCTAACGAAATGCAA | TCCATCAGAGGCTCCTTCTT | 127 | 104.35 |
| *DUR1,2* | urea carboxylase/allophanate hydrolase | GGAGTTATGGGATGTTCCGT | ATTATAGGGCCGACACCAAC | 72 | 103.96 |
| *dapB* | 4-hydroxy-tetrahydrodipicolinate reductase | CACCACCGGATGAAGTTAGA | CAATTTCGCCCGATAGTCTC | 136 | 100.9 |
| 1. qRT-PCR for *Portiera* genes | |  |  |  |  |
| *dapA* | dihydrodipicolinate synthase | TTCTGTAATAGCGGGAATTGG | TCCCATATTGAGTTGGCTTG | 131 | 104.16 |
| *dapB* | dihydrodipicolinate reductase | GTTAGTAGGTGCCGTAA | TATTGCCAACTGGTTTT | 101 | 107.77 |
| *dapD* | 2,3,4,5-tetrahydropyridine-2-carboxylate N-succinyltransferase | CCCTCAAATGACAGATT | CCTAAGTAAGCACCTAAAC | 81 | 105.26 |
| *dapE* | succinyl-diaminopimelate desuccinylase | TGTTGGTCATACAGATGTAGTTCCT | CCTTTCATATCGACAATTCCAC | 120 | 100.53 |
| *argF* | ornithine carbamoyltransferase | TTGAAGATCCTTATCTTGCTGTAAA | TTCACACCATACCTCTTCGC | 92 | 107.62 |
| *argG* | argininosuccinate synthase | TTTAGGTAGTAAACATGGGATTGG | GATTCTATTGCTCTATGTGCATGAT | 129 | 102.31 |
| *argH* | argininosuccinate lyase | TTACAATCATCACAGCACA | ATTATTTTCCGCCAATC | 105 | 91.02 |
| *ilvA* | threonine dehydratase | AGGCACTTTAGCGATGGAAG | TGGCTCTACACCAACGATCT | 148 | 95.76 |
| *ilvH* | acetolactate synthase I/III small subunit | ACAAATCACTGGAGATGAAGATAAA | TTTACCCAAACACGCGGTA | 108 | 97.95 |
| *ilvI* | acetolactate synthase I/II/III large subunit | TGGTGGAGCAATAACAGGAA | CATCCCTGGCCAACCTAT | 139 | 103.64 |
| *ilvC* | ketol-acid reductoisomerase | GCAAGTGCTATTGGATGTGG | TCAAATCCAGCTGTTATTAATGC | 131 | 101.08 |
| *ilvD* | dihydroxy-acid dehydratase | CTAAAGCGCCAGGACATACA | ACATCCAATAGCACTTGCATAAG | 134 | 97.72 |
| 1. qRT-PCR reference genes | |  |  |  |  |
| *RPL13* | 60S ribosomal protein L13a | GTTCCCTGTGCTATGAGGGT | TCCAACCAACATCAGAGGAA | 85 | 105.73 |
| *RPL7* | 60S ribosomal protein L7a | TTTGCAGACGTCAGTTCTCC | TTACTGCCTTCTTCGGCTTT | 142 | 104.47 |

**Table S2 Metabolism gene content of *Portiera* with sequenced genomes (A-B) and requirements of *Portiera* and whitefly host for cofactors (C).** Details of *Portiera* genomes are provided in Supplemental Database S1

| **Table S2A** Genes of different metabolism classes | | | | | |
| --- | --- | --- | --- | --- | --- |
|  | *Number of genes* | | | | |
| *Metabolism class* | *Portiera BT-B1* | *Portiera BT-B2* | *Portiera BT-Q1* | *Portiera BT-Q2* | *Portiera TV* |
| Essential amino acid synthesis | 50 | 50 | 50 | 50 | 51 |
| Oxidative phosphorylation | 26 | 26 | 26 | 26 | 26 |
| Glycolysis | 4 | 4 | 4 | 4 | 4 |
| Pentose phosphate pathway | 3 | 3 | 3 | 3 | 3 |
| Carotenoid biosynthesis | 3 | 3 | 3 | 3 | 3 |
| TCA cycle | 2 | 2 | 2 | 2 | 2 |
| Peptidases | 2 | 2 | 2 | 2 | 2 |
| Cofactor synthesis | 2 | 2 | 2 | 2 | 2 |
| Purine metabolism | 5 | 5 | 5 | 5 | 5 |
| Glycerophospholipid metabolism | 1 | 1 | 1 | 1 | 1 |
| Selenoamino acid metabolism | 1 | 1 | 1 | 1 | 1 |
| Total | **99** | **99** | **99** | **99** | **100** |

| **Table S2B** Essential amino acid synthesis genes with number of pseudogenes indicated in parentheses | | | | | | |
| --- | --- | --- | --- | --- | --- | --- |
| *EAAs* | *Number of genes including pseudogenes*  *(number of pseudogenes in parentheses)* | | | | | *EAA biosynthesis genes present/absent* |
|  | *Portiera BT-B1* | *Portiera BT-B2* | *Portiera BT-Q1* | *Portiera BT-Q2* | *Portiera TV* |  |
| Arginine | 6 (1) | 6 (1) | 6 (1) | 6 (1) | 6 | *argDFGH*, *carAB*; *argABCE* missing; *argH* is pseudogene in *Portiera* BT |
| Histidine | 8 | 8 | 8 | 8 | 7 | *hisGZEIAFBC*; *hisD* missing; *Portiera*TV lacks *hisE* (this reaction is also mediated by *hisI*) |
| Isoleucine | 5 | 5 | 5 | 5 | 5 | *ilvAHICD*; *ilvE* missing |
| Leucine | 8 | 8 | 8 | 8 | 8 | *ilvHICD* and *leuABCD*;  *ilvE* missing |
| Lysine | 7 (1) | 7 (1) | 7 (1) | 7 (1) | 9 | *lysC*, *asd*, *dapABDEF*, *argD*, *lysA* in *Portiera* TV; in *Portiera* BT, *dapF* and *lysA* are missing and *dapB* is pseudogene |
| Methionine | 1 | 1 | 1 | 1 | 1 | *metE*; *metABC* missing |
| Phenylalanine | 8 | 8 | 8 | 8 | 8 | *aroABCEFGHKQ*, *pheA*; *tyrB* missing |
| Threonine | 5 | 5 | 5 | 5 | 5 | *thrABC*, *asd*, *lysC* |
| Tryptophan | 14 | 14 | 14 | 14 | 14 | *aroABCEFGHKQ*, *trpABCDEFG* |
| Valine | 4 | 4 | 4 | 4 | 4 | *ilvHICD*; *ilvE* missing |
| Total | **50** | **50** | **50** | **50** | **51** |  |

**Table S2C Requirements of *Portiera* and whitefly host for cofactors**

| *Cofactor* | *Portiera requirements* | *Host capability* |
| --- | --- | --- |
| Thiamine diphosphate and thiamine phosphate | For pyruvate decarboxylase  And 2-oxoglutarate dehydrogenase E1 component | Thiamine pyrophosphokinase for thiamine to thiamine diphosphate; nucleoside-triphosphatase for thiamine diphosphate to thiamine phosphate |
| Heme and cytochrome c | [for electron transport chain](http://en.wikipedia.org/wiki/Electron_transport_chain) | Contain heme synthesis enzymes |
| Ubiquinone-n | [for electron transport chain](http://en.wikipedia.org/wiki/Electron_transport_chain) | Contain ubiquinone synthesis enzymes |
| FMN/FADH2 | For pyruvate decarboxylase and MetF | Can transform riboflavin to FMN and FADH2 |
| NAD(P)H | Nicotinamide required for redox reactions | Enzymes for transforming nicotinate and nicotinamide to NAD, nicotinamide riboside kinase, NAD+ kinase and NAD+ synthase |
| Pantothenate | None identified | No capacity to synthesize pantothenate, but contain CoA synthesis enzymes |
| Pyridoxal-5’-phosphate (PLP) | For various amino acid biosynthesis reactions | Pyridoxamine 5'-phosphate oxidase is enriched in bacteriocytes |
| Biotin | None identified | HTGs bioAB. Bacteriocyte may need it for its fatty acid synthesis. |
| Folic acid | THF required for methionine synthesis | Can synthesize THF from 7,8-dihydropteroate |

Notes: *Hamiltonella* has de novo capability to synthesize riboflavin, heme, NAD(P), PLP, ubiquinone, CoA and biotin, and probably needs all of these for its own metabolism. *Hamiltonella* lacks the genetic capacity to synthesize thiamine and pantothenate

**Table S3 Summary statistics of whitefly RNA-Seq**

| *Category* | *Value for mRNA-Seq library* | |
| --- | --- | --- |
|  | *Bacteriocyte* | *Whole body* |
| Total Raw Reads | 55,421,140 | 61,200,096 |
| Total Clean Reads | 52,385,202 | 51,798,210 |
| Total Contig Number | 97,534 | 120,412 |
| Total Contig Length(nt) | 51,299,889 | 47,126,408 |
| Total Unigene Number | 51,479 | 68,972 |
| Total Unigene Length(nt) | 67,638,283 | 46,081,180 |
| Distinct Singletons | 36,143 | 55,236 |

**Table S4 qPCR and RT-PCR results**

**Table S4A** qRT-PCR validation of RNA-seq data for selected *Bemisia* genes. The fold-difference in expression between bacteriocytes and whole body (log_2_) scale, relative to two normalizing genes (RP7 and RP13) is displayed (mean + s.e., 3 biological replicates)

| *Gene name* | *Description* | *Log_2_ Ratio _RNA-seq* | *Log_2_ Ratio _qPCR_RP7* | |  | *Log_2_ Ratio _qPCR_RP13* | |
| --- | --- | --- | --- | --- | --- | --- | --- |
|  |  |  | *Mean* | *s.e* |  | *Mean* | *s.e* |
| *GOGAT* | glutamate synthase | 2.047 | 2.007 | 0.248 |  | 1.104 | 0.192 |
| *aspC* | aspartate aminotransferase | 1.728 | 2.156 | 0.123 |  | 2.574 | 0.113 |
| *BCAT* | branched-chain-amino-acid aminotransferase | 1.581 | 5.466 | 0.107 |  | 4.563 | 0.147 |
| *serA* | D-3-phosphoglycerate dehydrogenase | 3.947 | 5.28 | 0.056 |  | 5.693 | 1.205 |
| *atoB* | acetyl-CoA C-acetyltransferase | 1.817 | 0.948 | 0.125 |  | 1.229 | 0.451 |
| *FDPS* | farnesyl diphosphate synthase | 1.732 | 1.353 | 0.173 |  | 0.517 | 0.040 |
| *MVK* | mevalonate kinase | 2.670 | 5.082 | 0.759 |  | 3.372 | 0.132 |
| *SDR16C5* | all-trans-retinol dehydrogenase | 3.744 | 5.514 | 0.119 |  | 4.223 | 0.023 |
| *fabD* | [acyl-carrier-protein] S-malonyltransferase | 1.121 | 2.900 | 0.764 |  | 1.190 | 0.179 |
| *PECR* | trans-2-enoyl-CoA reductase | 1.440 | 1.471 | 0.802 |  | 0.884 | 0.183 |
| *FAD6* | delta-12 desaturase | 1.247 | 2.769 | 0.215 |  | 1.911 | 0.165 |
| *Psd* | phosphatidylserine decarboxylase | 1.073 | 3.151 | 0.052 |  | 2.430 | 0.056 |
| *GPAT* | glycerol-3-phosphate O-acyltransferase 3/4 | 1.372 | 3.764 | 1.118 |  | 1.239 | 0.081 |
| *HADHA* | enoyl-CoA hydratase | 1.154 | 0.978 | 0.130 |  | 2.452 | 0.376 |
| *HMG-CoA reductase* | hydroxymethylglutaryl-CoA reductase (NADPH) | 1.773 | 1.531 | 0.267 |  | 1.622 | 0.479 |
| *argH* | argininosuccinate lyase | 1.417 | 4.397 | 0.244 |  | 3.596 | 0.191 |
| *lysA* | diaminopimelate decarboxylase | 3.806 | 5.311 | 0.085 |  | 4.509 | 0.087 |
| *dapF* | diaminopimelate epimerase | 0.940 | 5.753 | 0.325 |  | 4.951 | 0.379 |
| *CM* | chorismate mutase | 0.971 | 2.736 | 0.273 |  | 1.881 | 0.210 |
| *PDHA1* | pyruvate dehydrogenase | -2.704 | -6.398 | 1.196 |  | -5.231 | 0.243 |
| *PDHA1* | pyruvate dehydrogenase | -1.847 | -0.841 | 0.040 |  | -1.696 | 0.043 |
| *DUR1,2* | urea carboxylase/allophanate hydrolase | 0.800 | 3.455 | 0.431 |  | 2.384 | 0.177 |
| *bioA* | adenosylmethionine-8-amino-7-oxononanoate aminotransferase | 0.723 | 1.719 | 0.157 |  | 0.648 | 0.032 |
| *dapB* | 4-hydroxy-tetrahydrodipicolinate reductase | 3.804 | 4.775 | 0.188 |  | 3.704 | 0.095 |
| *bioB* | biotin synthase | 0.120 | 0.265 | 0.156 |  | -0.806 | 0.155 |
| *AH* | allophanate hydrolase | 2.870 | 5.045 | 0.144 |  | 3.974 | 0.021 |
| *argG* | argininosuccinate synthase | 0.788 | 3.436 | 0.159 |  | 2.365 | 0.067 |

**Table S4B** qRT-PCR analysis of *Portiera* gene expression. The transcript abundance (2^-△Ct^) of genes in lysine, isoleucine or arginine synthesis pathways, relative to two normalizing genes (RP7 and RP13) is displayed (mean + s.e., 3 biological replicates)

| *Gene name* | *Description* | *Ratio _qPCR_RP7* | |  | *Ratio _qPCR_RP13* | |
| --- | --- | --- | --- | --- | --- | --- |
|  |  | *Mean* | *s.e* |  | *Mean* | *s.e* |
| (a) lysine synthesis pathway | |  |  |  |  |  |
| *dapA* | dihydrodipicolinate synthase | 1.585 | 0.100 |  | 0.298 | 0.008 |
| *dapB* | dihydrodipicolinate reductase | 0.003 | 0.001 |  | 0.001 | 0.000 |
| *dapD* | 2,3,4,5-tetrahydropyridine-2-carboxylate N-succinyltransferase | 0.222 | 0.043 |  | 0.041 | 0.007 |
| *dapE* | succinyl-diaminopimelate desuccinylase | 1.107 | 0.087 |  | 0.208 | 0.009 |
| (b) arginine synthesis pathway | |  |  |  |  |  |
| *argF* | ornithine carbamoyltransferase | 1.581 | 0.030 |  | 0.299 | 0.012 |
| *argG* | argininosuccinate synthase | 0.712 | 0.071 |  | 0.134 | 0.008 |
| *argH* | argininosuccinate lyase | 0.024 | 0.005 |  | 0.005 | 0.001 |
| (c) isoleucine synthesis pathway | |  |  |  |  |  |
| *ilvA* | threonine dehydratase | 0.606 | 0.038 |  | 0.113 | 0.009 |
| *ilvH* | acetolactate synthase I/III small subunit | 3.139 | 0.196 |  | 0.584 | 0.047 |
| *ilvI* | acetolactate synthase I/II/III large subunit | 2.951 | 0.087 |  | 0.548 | 0.025 |
| *ilvC* | ketol-acid reductoisomerase | 7.507 | 0.702 |  | 1.398 | 0.157 |
| *ilvD* | dihydroxy-acid dehydratase | 6.583 | 0.472 |  | 1.225 | 0.111 |
